# The HER Salt Lake media campaign: comparing characteristics and outcomes of clients who make appointments online versus standard scheduling

**DOI:** 10.1186/s12905-021-01256-x

**Published:** 2021-03-23

**Authors:** Kyl Myers, Jessica N. Sanders, Cristen Dalessandro, Corinne D. Sexsmith, Claudia Geist, David K. Turok

**Affiliations:** 1 Division of Family Planning, Department of Obstetrics and Gynecology, University of Utah School of Medicine, 30 N 1900 E, 2B200, Salt Lake City, UT 84132 USA; 2 Department of Sociology and Division of Gender Studies, University of Utah, Salt Lake City, USA

**Keywords:** Contraceptives, Media campaign, Contraceptive initiative, LARCs

## Abstract

**Background:**

Little research has examined how media outreach strategies affect the outcomes of contraceptive initiatives. Thus, this paper assesses the potential impact of an online media campaign introduced during the last six months of a contraceptive initiative study based in Salt Lake City, UT (USA).

**Methods:**

During the last six months of the HER Salt Lake Contraceptive Initiative (September 2016-March 2017), we introduced an online media campaign designed to connect potential clients to information about the initiative and a brief (9-item) appointment request form (via HERsaltlake.org). Using linked data from the online form and electronic medical records, we examine differences in demographics, appointment show rates, and contraceptive choices between “online requester” clients who made clinical appointments through the online form (n = 356) and “standard requester” clients who made appointments using standard scheduling (n = 3,051). We used summary statistics and multivariable regression to compare groups.

**Results:**

The campaign logged 1.7 million impressions and 15,765 clicks on advertisements leading to the campaign website (HERSaltLake.org). Compared to standard requesters, online requesters less frequently reported a past pregnancy and were more likely to be younger, white, and to enroll in the survey arm of the study. Relative to standard requesters and holding covariates constant, online requesters were more likely to select copper IUDs (RRR: 8.14), hormonal IUDs (RRR: 12.36), and implants (RRR: 10.75) over combined hormonal contraceptives (the contraceptive pill, patch, and ring). Uptake of the contraceptive injectable, condoms, and emergency contraception did not differ between groups.

**Conclusion:**

Clients demonstrating engagement with the media campaign had different demographic characteristics and outcomes than those using standard scheduling to arrange care. Online media campaigns can be useful for connecting clients with advertised contraceptive methods and initiatives. However, depending on design strategy, the use of media campaigns might shift the demographics and characteristics of clients who participate in contraceptive initiatives.

**Trial Registration:**

Clinicaltrials.gov identifier NCT02734199, Registered 12 April 2016—Retrospectively registered, https://clinicaltrials.gov/ct2/show/NCT02734199.

**Supplementary Information:**

The online version contains supplementary material available at 10.1186/s12905-021-01256-x.

## Background

In recent years, several states and academic institutions in the U.S. have introduced contraceptive initiatives—or, programs designed to reduce contraceptive cost and access barriers, especially around intrauterine devices (IUDs) and implants. These initiatives have generally been successful at increasing public awareness and uptake of contraceptives and in reducing rates of unintended pregnancies, especially among underserved groups [1–4]. However, the extent to which advertising and recruitment strategies matter for the outcomes of these initiatives remains understudied. While some research has shown that advertising may not make a marked difference in contraceptive uptake more generally [5], we lack information regarding the potential impact of advertising on contraceptive initiatives offering no-cost contraception to prospective clients.

Generally, when it comes to campaigns or initiatives related to health promotion, past research has consistently found that media outreach can be an effective strategy for increasing health-related knowledge among the public and improving health outcomes [6–10]. Research also supports the use of online media strategies in reaching specific, elusive groups, as well as those living in socially conservative contexts [11]. In the past, researchers have found word-of-mouth advertising to be successful in recruiting participants for contraceptive initiatives [12]. However, certain groups of potential clients might not be reached as easily by word-of-mouth. For example, in the United States, research has found that social media and online advertising are especially effective at delivering sexual health and contraceptive information to younger adults (age 18–29), who often look for guidance online [13–16]. Since young adults also have the highest rates of unplanned pregnancy in the United States [17], they are an important potential audience for contraceptive initiatives.

Regarding sexual and reproductive health in particular, past research has found that incorporating media campaigns and online platforms providing contraceptive information (such as Bedsider.org) into research may impact how participants perceive certain methods (especially long-acting reversible contraception, or LARCs) and individuals’ contraceptive choices [18–20]. Information on who participates (and who may be left out) as a result of advertising or media outreach efforts, as well as investigating how the use of media may impact client choices, is especially important for informing contraceptive initiative recruitment efforts and in understanding how media messages might matter for clients’ contraceptive choices.

In light of the potential for media outreach or advertising to make a difference in the outcomes of contraceptive initiatives, this paper explores the outcomes of an online media campaign introduced during the latter third of the HER Contraceptive Initiative Study (HER Salt Lake) based in Salt Lake City, Utah (USA). As part of the media campaign, advertising directed prospective initiative clients to a simple, nine item online form through the campaign website (HERSatltLake.org) in order to set up appointments for contraceptive care. We compare the clients who made appointments at Planned Parenthood Association of Utah (referred to hereafter as “PPAU”) clinics via the online form designed for the media campaign (“online requesters”) to clients who did not make their appointments via the HER Salt Lake online form (“standard requesters”). We investigate demographic and characteristic differences between the two groups of clients as well as differences in appointment show rates and method uptake choices. Based on previous research, we hypothesize that the clients reached during our media campaign will be younger and more likely to choose LARC methods than those served during the first two periods of the study, which did not utilize a media campaign. Ultimately, our findings suggest that advertising media campaigns may make a difference when it comes to the demographic characteristics and outcomes of clients participating in a contraceptive initiative.

## Methods

### Design

HER Salt Lake increased community-wide access to contraceptives over the course of 12 months (March 2016–March 2017) by removing contraceptive service costs for eligible clients at four participating PPAU health centers. Contraceptive options that clients could access free of cost included: emergency contraception; contraceptive pills, patches, rings and injections; hormonal and non-hormonal IUDs; contraceptive implants; condoms; and diaphragms. Additionally, clients could switch methods at no cost for up to three years after their first visit. A detailed description of the project and changes in method uptake among clients is reported elsewhere [4]. The Institutional Review Board at the authors’ institution approved the protocol for the project, including the plans for the media campaign.

Project data collection occurred during three periods: an initial control period, or Period 1 (September 28, 2015 through March 27, 2016); Period 2 (March 28, 2016 through September 25, 2016), which provided increased access and no-cost care; and Period 3 (September 26, 2016 through March 25, 2017), which continued increased access and no-cost care with an added media campaign. During all three periods, clients served at participating clinics who were 18 and over were eligible to enroll in a 3-year prospective survey, and 4,425 enrolled. The survey completed by those enrolled is provided in Additional file 1 (“Enrollment Survey”). In this paper, we describe the online media campaign, engagement, and outcomes including scheduling and method uptake.

During Period 3, the research team partnered with a digital marketing agency to build and launch the campaign, including an official campaign website (HERSaltLake.org) as well as Facebook and Instagram pages. We designed the electronic media campaign to reach the handheld devices of cisgender, nonbinary, and transgender individuals 18- to 34-years-old with potential need for contraceptive services. The research team also increased media presence by participating in local and national press interviews and by writing commentary pieces during the media campaign, which were available online [21, 22]. Due to the marketing agency’s social commitment to the local metropolitan community, they offered a number of services to the HER Salt Lake team at a discounted price. Ultimately, the campaign put $10,000 towards the creation and monitoring of HERSaltLake.org and $20,000 towards purchasing targeted advertisements and keyword search results, all managed by the marketing agency.

**Fig. 1:**
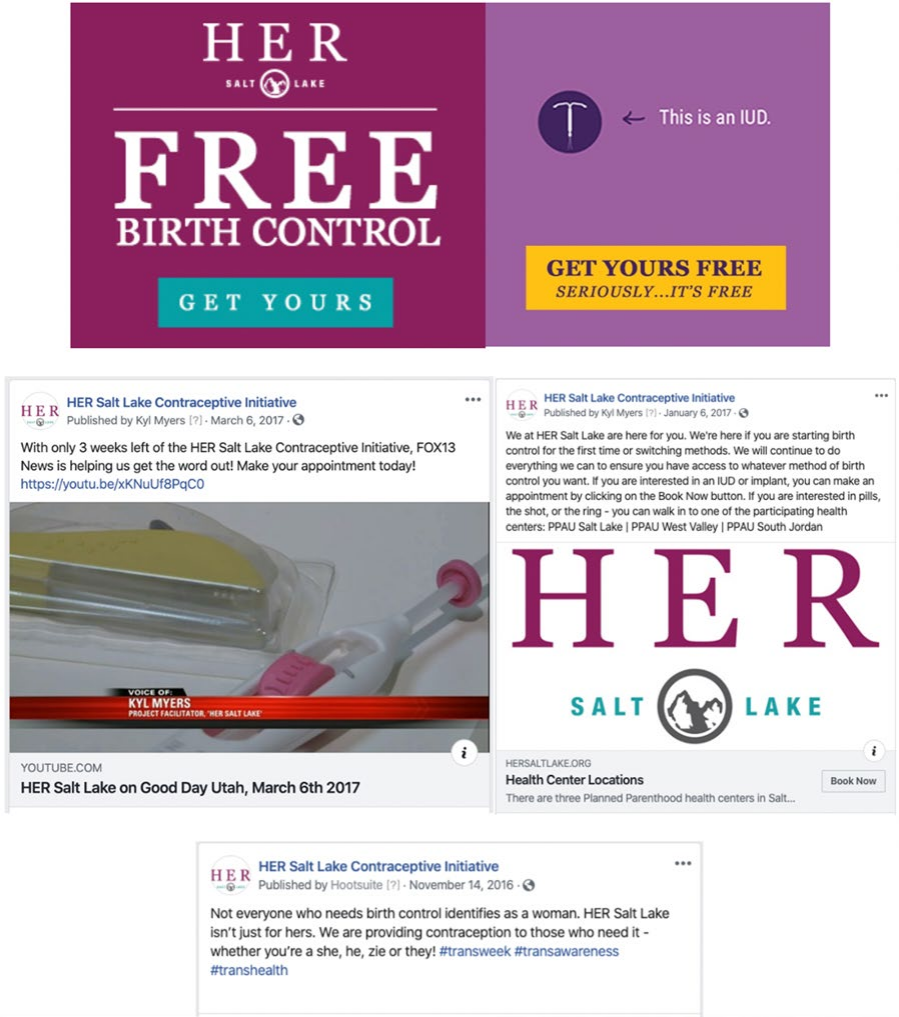
Example advertisements from the HER Salt Lake Period 3 media campaign

The campaign website had information about available contraceptive methods and linked to Bedsider.org’s method finder tool to help interested individuals learn more about different contraceptives [23]. Past research has shown that individuals seeking contraceptive care or information about sexual health view Bedsider.org positively and consider it a trustworthy resource [24, 25]. Individuals visiting Bedsider.org who had an IP address placing them in Utah received a pop-up letting them know about no-cost contraception in their area and a link to the HERSaltLake.org campaign website. The *Locations* webpage on HERSaltLake.org listed the addresses and phone numbers for PPAU health centers accepting appointments in the county. The *Locations* page also stated that individuals could walk into any participating health center and receive same-day services for emergency contraception, oral contraceptive pills, patches, rings, injections, and condoms. For those interested in an IUD or implant, the website provided two options: clients could call a scheduling center and make an appointment, or submit a simple nine-question appointment request form located on the webpage. The media strategy also included geo-targeted paid search ads for HERSaltLake.org, which appeared as the top search result when individuals in the county utilized a search engine with specific keywords (see Table 1). Further, we posted advertisements and creative content on the Facebook and Instagram pages linking to HERSaltLake.org (see Fig. 1). Advertisements used modified creative content developed for WhoopsProof.org, a program of Power to Decide (which also operates Bedsider.org), and original content created by the project communications team. All calls to action on advertisements led to the *Locations* page on HERSaltLake.org.

**Table 1:** Media campaign search keywords by advertisement group

IUD group	Implant group	Birth control options group
IUD	The Implant	Birth Control
Mirena Price	Contraceptive Implant	Best Birth Control
Contraceptive IUD	Birth Control Implant	Methods of Birth Control
IUD Birth Control	The Implant Contraception	Free Birth Control Utah
Copper Intrauterine Device IUD	Birth Control Implant Cost	How to Get Birth Control Without Insurance

### Sample data

To assess the reach of the media campaign, we utilized three data sources: media campaign analytics, the online appointment request form accessed through HERSaltLake.org, and Electronic Health Records (EHRs) from PPAU health centers. Media campaign analytic reports served as our first source of data. The digital marketing agency compiled these reports, which provided information on the reach of the media campaign such as the number of impressions and engagements with the campaign (measured by individuals “clicking” on sponsored links).

Our second source of data, the online appointment request form, asked for nine pieces of information including name, date of birth, current method of contraception, desired method of contraception, preferred appointment date, preferred appointment time, preferred appointment location, phone number, and email (see Table 2). This differs from the online appointment request form traditionally used by PPAU, which requests the completion of up to 41 information fields. We intentionally limited the number of items to minimize the time needed to complete forms [26].

**Table 2:** Online appointment request form

Field	Data Format	Rationale
Name	Client entered First and Last name	To set an appointment and establish if the client is a new or returning patient
Date of Birth	Client entered MM/DD/YYYY	To verify age eligibility for initiative (16–45 years)
Current method of contraception	Selected current method from drop-down menu: condoms; IUD; implant; pill; patch; ring; shot; none; other	To verify eligibility for a walk-in appointment or need for an appointment with a provider
Desired method of contraception	Client selected desired method from drop-down menu: condoms; IUD; implant; pill; patch; ring; shot; none; other	To verify eligibility for a walk-in appointment (user-dependent method) or need for an appointment with a provider (IUD or implant removal/insertion) and also to verify eligibility for no-cost contraception through the contraceptive initiative study i.e. starting or switching a method
Preferred appointment date	Client entered MM/DD/YYYY of preferred appointment date	Scheduling staff would make every effort to schedule an appointment for the date preferred by the potential client. Clinic business days varied by location
Preferred appointment time	Client entered time and A.M./P.M. of preferred appointment	Scheduling staff would make every effort to schedule an appointment for the time preferred by the potential client. Clinic business hours varied by location
Preferred appointment location	Client selected location from drop down menu of three clinic options	Scheduling staff would make every effort to schedule an appointment for the location preferred by the potential client
Phone number	Client entered phone number XXX-XXX-XXXX	Scheduling staff contacted potential clients within 24 h or the next business day to confirm a requested appointment or to find another time if the requested appointment was not possible
E-mail	Client entered e-mail address	Scheduling staff contacted potential clients within 24 h or the next business day to confirm a requested appointment or to find another time if the requested appointment was not possible

Clients’ online appointment request forms were sent to one, centralized PPAU scheduling e-mail address monitored by PPAU scheduling staff. For potential clients not currently using an IUD or implant (requiring removal) and interested in pills, patches, rings, injections or condoms, staff contacted individuals to inform them that they could walk-in to any participating clinic and be seen without an appointment or schedule an appointment if preferred. Among potential clients currently using an IUD or implant or interested in obtaining an IUD or implant, scheduling staff booked an appointment with a provider. The staff prioritized accommodating preferred appointment request dates, times, and locations of online requesters. It was not uncommon to double book appointments. If unable to accommodate an appointment request (due to preferred appointment being after business hours or no providers in clinic that day), the staff member would contact the potential client and find another satisfactory appointment option. In all cases, PPAU staff members attempted to contact potential clients to confirm appointment requests or discuss options.

**Fig. 2:**
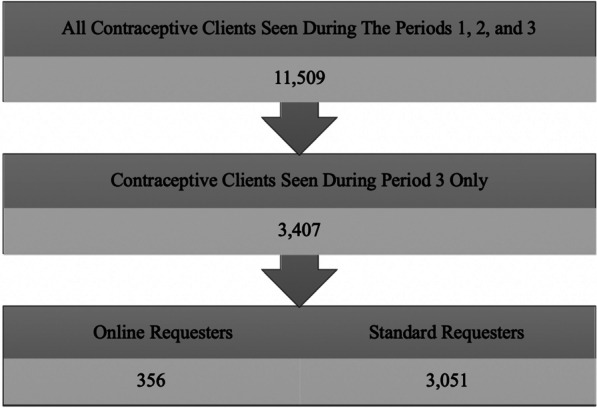
Participant inclusion/exclusion flow chart in context of larger contraceptive initiative study

Our third source of data comes from PPAU’s EHR. PPAU uses NexGen Healthcare (Irvine, CA) to manage client information, including demographics and medical history. We used EHR data to collect data elements for both online requester clients and standard requester clients who sought contraceptive care during Period 3. Data elements included: patient ID, information on if a client’s first visit occurred at PPAU during the media campaign and corresponding date, age, race/ethnicity, pregnancy history, whether clients enrolled in the survey portion of the project, location of visit, and birth control outcomes. We also explored whether clients showed up for scheduled appointments and, if applicable, rescheduled appointments. We used EHRs to follow up on whether clients visited one of the clinics after engagement with the media campaign (filling out an appointment request online) and compared online requester clients to eligible standard clients visiting the clinic during the media campaign who did not make their appointments via the online form. Standard requesters used standard scheduling—methods already used by PPAU, including direct calls to clinics and completing the longer online form. Clinical research staff aligned online request forms by patient identifiers with electronic health record data to generate data on demographics and outcomes using REDCap, a secure web platform for designing and managing online databases [27]. Staff checked client IDs to ensure no duplicates. Figure 2 details the participant flow in the context of the larger contraceptive initiative study. Compared to Periods 1 and 2, clients served in Period 3 account for approximately 30% of the total number of clients served during the entire contraceptive initiative.

### Analysis

We conducted descriptive statistics assessing characteristics of online requesters and standard requesters. First, we used chi-square tests of association to compare the demographic characteristics of the two groups. To conduct our analysis, we excluded participants who initiated care during Periods 1 and 2 of the study so as to assess only those whose initial visit occurred while the media campaign was live. Additionally, in the event that individual clients submitted multiple online request forms, we excluded duplicates in order to count each client only once as an online requester in our analysis. Next, we used multinomial logistic regression to compare the contraceptive choices of standard requester and online requester clients in Period 3 (during the media campaign). We also examined information about when potential clients submitted requests and the location of desired appointments. In a last analytic step, we used multinomial logistic regression to explore the types of contraceptive methods selected by those who used the online scheduling system compared to the standard requester clients. We analyzed the combined dataset in Stata version 16 [28].

## Results

From September 28, 2016 to March 25, 2017, there were approximately 1.7 million impressions of the campaign’s paid advertisements and 15,765 clicks on advertisements leading to HERSaltLake.org. Ultimately, clients and potential clients submitted 891 online appointment requests through the website. Our form received more than three times the amount of appointment requests compared to PPAU’s longer online form (they report receiving approximately 40 forms per month). PPAU staff was able to follow up and establish contact with 95% of those who filled out an online form from HERSaltLake.org.

**Fig. 3:**
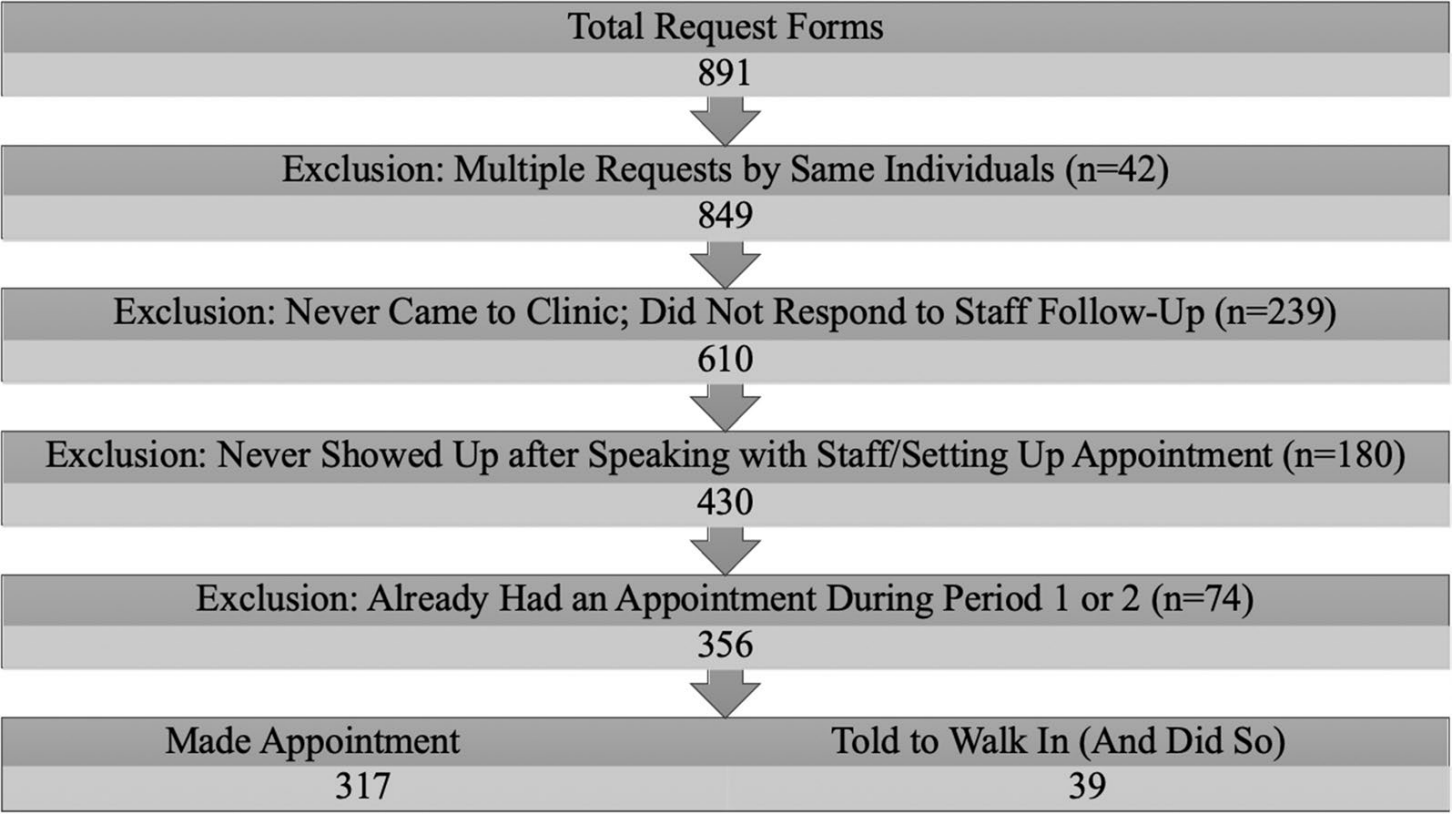
Sample inclusion/exclusion flow chart—online requesters

Excluding duplicate requests and potential clients who did not respond to PPAU staff follow-up, a total of 610 online requesters either made appointments or were advised by staff to present to the clinic as walk-ins. Approximately 30% of these potential clients (n = 180) never showed up to clinic. After also excluding those who were already enrolled in the study prior to Period 3 and were using the website to make a follow-up appointment, a total of 356 new clients arranged and eventually received care as an outcome of completing the online enrollment form. Of these clients, staff advised 39 to walk in (and did so), while 317 made appointments. Figure 3 details participant flow. Approximately 72% (n = 257) of the appointment requests for these 356 clients came in between the hours of 9 am and 8 pm, with 89% (n = 315) expressing interest in an IUD or implant. While 51 (or 16%) of the 356 clients did not show up to their first appointment, these 51 rescheduled appointments and subsequently did show. Half (50%) of online requester clients were completely new to Planned Parenthood and had never been to a PPAU clinic prior.

In examining the demographics of the online requesters and standard requesters, a greater percentage of online requesters were between 18 and 34 years of age, were more racially white, and were more likely to enroll in the survey portion of the contraceptive initiative study. Online requesters reported less incidence of pregnancy. In addition, online requesters favored certain clinic sites, and choose IUDs or implants more frequently than standard requesters. Table 3 summarizes these demographic differences.

**Table 3:** Demographic characteristics comparing the “online requesters” to the “standard requesters” cohort (September 26, 2016—March 25, 2017)

	Standard requesters	Online requesters	*p*
	n (%)	n (%)	
*Age*			
< 18	205 (7)	2 (1)	< 0.001
18–24	1431 (47)	207 (58)	
25–34	1035 (34)	131 (37)	
35 +	380 (12)	16 (4)	
*Race/Ethnicity*			
White Non-Hispanic	1889 (62)	255 (72)	< 0.001
Hispanic	838 (27)	57 (16)	
Other	324 (11)	44 (12)	
*Insurance status*			
No Insurance	2082 (68)	229 (64)	0.135
Insurance	969 (32)	127 (36)	
*Past pregnancy*			
No	1968 (65)	275 (77)	< 0.001
Yes	1083 (35)	81 (23)	
*HER study enrollment*			
No	1924 (63)	82 (23)	< 0.001
Yes	1127 (37)	274 (77)	
*Clinic location*			
Metro Health Clinic	241 (8)	17 (5)	< 0.001
Salt Lake City Clinic	991 (32)	158 (44)	
South Jordan Clinic	696 (23)	79 (22)	
West Valley Clinic	1123 (37)	102 (29)	
*Birth control outcomes*			
Condoms, Diaphragm, Emergency Contraception	216 (7)	5 (1)	< 0.001
Contraceptive Shot	425 (14)	16 (4)	
Contraceptive Implant	283 (9)	91 (26)	
Copper IUD	243 (8)	59 (17)	
Hormonal IUD	434 (14)	149 (42)	
None	177 (6)	1 (< 1)	
Pill, Patch, or Ring	1273 (42)	35 (10)	

We also performed multinomial logistic regression to assess differences in contraceptive choices among the online requesters and standard requesters. Controlling for other covariates, online requesters more often chose copper IUDs (RRR: 8.14), hormonal IUDs (RRR: 12.36), and implants (RRR: 10.75) compared to the birth control pill, patch, or ring. As shown in Table 4, they were also more likely to choose these methods compared to those in the standard requesters cohort.

**Table 4:** Multinomial logistic regression of client contraceptive choices

Condoms, Diaphragm, EC	Relative Risk Ratio	95% CI	Copper IUD	Relative Risk Ratio	95% CI	Levonorgestrel IUD	Relative Risk Ratio	95% CI
*Client group*			*Client group*			*Client group*		
Online Requesters	1.265	[0.480; 3.331]	Online Requesters	**8.141†**	[5.155; 12.856]	Online Requesters	**12.364†**	[8.261; 18.503]
*Age*			*Age*			*Age*		
< 18	1	1	< 18	1	1	< 18	1	1
18–24	2.633*	[1.231; 5.632]	18–24	3.189	[0.981; 10.367]	18–24	0.807	[0.445; 1.464]
25–34	**5.846†**	[2.711; 12.603]	25–34	4.483*	[1.368; 14.695]	25–34	0.984	[0.535; 1.811]
35 +	**14.137†**	[6.152; 32.486]	35 +	**6.655‡**	[1.940; 22.833]	35 +	1.169	[0.589; 2.318]
*Race/ethnicity*			*Race/Ethnicity*			*Race/ethnicity*		
White	1	1	White	1	1	White	1	1
Hispanic	1.047	[0.722; 1.519]	Hispanic	0.943	[0.663; 1.342]	Hispanic	0.885	[0.663; 1.182]
Other	0.655	[0.379; 1.134]	Other	0.954	[0.630; 1.443]	Other	0.789	[0.550; 1.131]
*Insurance*			*Insurance*			*Insurance*		
Yes	0.890	[0.645; 1.228]	Yes	**2.124†**	[1.597; 2.826]	Yes	**2.202†**	[1.730; 2.802]
*Ever been pregnant*			*Ever been pregnant*			*Ever been pregnant*		
Yes	**0.342†**	[0.226; 0.516]	Yes	**1.643‡**	[1.194; 2.259]	Yes	**1.952†**	[1.495; 2.548]
*Clinic Location*			*Clinic location*			*Clinic location*		
clinic 1	1	1	Clinic 1	1	1	Clinic 1	1	1
Clinic 2	1.254	[0.368; 4.268]	Clinic 2	**0.216†**	[0.129; 0.363]	Clinic 2	**0.094†**	[0.0605; 0.147]
Clinic 3	1.816	[0.532; 6.199]	Clinic 3	**0.123†**	[0.070; 0.215]	Clinic 3	**0.087†**	[0.055; 0.138]
Clinic 4	1.364	[0.402; 4.628]	Clinic 4	**0.155†**	[0.0916; 0.263]	Clinic 4	**0.099†**	[0.064; 0.154]
*Study enrollment?*			*Study enrollment?*			*Study enrollment?*		
Yes	**0.163†**	[0.097; 0.274]	Yes	**2.518†**	[1.900; 3.338]	Yes	**3.878†**	[3.043; 4.941]

Contraceptive injectable	Relative risk ratio	95% CI	Contraceptive implant	Relative risk ratio	95% CI	None or missing methods	Relative risk ratio	95% CI
*Client group*			*Client group*			*Client group*		
Online requesters	1.630	[0.885; 3.002]	Online requesters	**10.753†**	[6.974; 16.579]	Online requesters	0.368	[0.050; 2.733]
*Age*			*Age*			*Age*		
< 18	1	1	< 18	1	1	< 18	1	1
18–24	**0.536**‡	[0.372; 0.771]	18–24	3.586*	[1.103; 11.659]	18–24	13.457*	[1.836; 98.648]
25–34	**0.350†**	[0.231; 0.531]	25–34	2.376	[0.719; 7.857]	25–34	**28.735**‡	[3.911; 211.129]
35 +	**0.415**‡	[0.247; 0.697]	35 +	1.999	[0.565; 7.070]	35 +	**83.972†**	[11.210; 628.996]
*Race/ethnicity*			*Race/ethnicity*			*Race/ethnicity*		
White	1	1	White	1	1	White	1	1
Hispanic	1.341*	[1.032; 1.743]	Hispanic	**2.246†**	[1.670; 3.021]	Hispanic	0.897	[0.597; 1.348]
Other	0.995	[0.685; 1.445]	Other	1.167	[0.778; 1.753]	Other	0.801	[0.457; 1.405]
*Insurance*			*Insurance*			*Insurance*		
Yes	0.809	[0.622; 1.051]	Yes	**2.083†**	[1.573; 2.757]	Yes	0.867	[0.602; 1.248]
*Ever been pregnant*			*Ever been pregnant*			*Ever been pregnant*		
Yes	**1.791†**	[1.354; 2.369]	Yes	**2.498†**	[1.852; 3.371]	Yes	0.855	[0.575; 1.273]
*Clinic location*			*Clinic location*			*Clinic location*		
Clinic 1	1	1	Clinic 1	**1**	1	Clinic 1	1	1
Clinic 2	1.148	[0.532; 2.477]	Clinic 2	**0.214†**	[0.125; 0.365]	Clinic 2	0.553	[0.226; 1.353]
Clinic 3	1.330	[0.613; 2.890]	Clinic 3	**0.161†**	[0.092; 0.283]	Clinic 3	0.574	[0.230; 1.428]
Clinic 4	1.766	[0.828; 3.770]	Clinic 4	**0.340†**	[0.204; 0.570]	Clinic 4	0.703	[0.293; 1.689]
*Study enrollment?*			*Study enrollment?*			*Study enrollment?*		
Yes	1.066	[0.828; 1.373]	Yes	**4.798†**	[3.631; 6.339]	Yes	**0.088†**	[0.041; 0.190]

*p < 0.05; ‡*p* < 0.01; †*p* < 0.001

Bold values denote statistically significant findings

## Discussion

We found notable demographic differences between the media campaign requester clients and standard requester clients. Compared to standard requesters, a higher percentage of online requesters were in the initiative’s targeted age range (18–34) and subsequently elected to enroll in the survey arm of the contraceptive initiative study. This age difference might be related to previous researchers’ finding that younger adults have confidentiality concerns—possibly due to being on their parents’ healthcare plans—that drive them to look online in order to investigate or arrange sexual and reproductive health care [29, 30]. We also posit that the online requesters may have differed from the standard requesters with regard to age due to the tendency of younger adults to use technology to seek health information [14, 15].

Online requesters were also more likely to receive IUDs and implants at their clinic appointments. Though this finding differs from previous assessments of media campaigns on LARC uptake [5], the offer of no-cost contraception during the media campaign had a probable role in increasing the likelihood that clients chose LARC methods. For example, the contraceptive initiative study project unfolded in three waves, and those clients in waves 2 and 3 who were offered no-cost contraception were more likely to choose LARC methods than those in the control period. Though they were both offered no-cost contraception, clients were more likely to choose LARC methods overall in Period 3 [4]. At the same time, those with the greatest likelihood of choosing LARC methods were those who made appointments through the media campaign’s request form. Differences in LARC uptake could thus be due in part to the availability of Bedsider.org as a resource offered to those completing the online form. Reading information on Bedsider.org may have increased client knowledge about LARCs, potentially piquing interest in LARC methods.

The design of our study makes it difficult to prove that standard requesters were not exposed to the media campaign. It is possible that some clients were exposed to the campaign and chose to request their appointment in a standard fashion anyway. However, those clients who were exposed to the campaign, engaged with it (measured by their completion of the 9-item form), and showed up to their appointments were both demographically different from the standard requester group *and* had different method choice outcomes. Our findings thus suggest that the importance of advertising strategies on contraceptive initiative campaigns—rather than making no difference—needs further attention. In the future, researchers incorporating media campaigns into their contraceptive initiative projects should note that such an inclusion could have an impact on *both* on the demographic characteristics of the participant sample *and* participants’ contraceptive choices. The use of media as well as the design of the media campaign itself (such as language featured in the campaign advertisements) can matter for sample demographics.

Also of note, our media campaign efforts did underrepresent some groups of potential clients who may have benefitted from participating in the contraceptive initiative. For example, if the campaign reached prospective clients equally, we would have expected comparable percentages of Hispanic clients and clinic location preference between the two study groups. However, both of these percentages were lower in the online requester group. This drop may be because our advertisements were only in English—a limitation of our study. Spanish advertisements may have changed the potential clients that our online advertisements reached, including more clients from the clinic in our study (Clinic 4) that typically serves the highest number of Spanish-speaking clients. Ads in both English and Spanish may have also better communicated the initiative’s commitment to serving Hispanic or bilingual members of the community. Additionally, in order to see the majority of the media campaign activities, potential clients would have to be online in the first place. Further, we did not devise a way to evaluate how (or if) the research team’s participation in local and national news or written commentary may have impacted the media campaign. It is possible to determine if these efforts drove some people to HERSaltLake.org where they completed the online form. Further, while we know who made their appointments using the media campaign’s online form, we also do not have details from the EHRs concerning which methods of booking appointments that the standard requesters used—phone call, filling out the PPAU form, walking in to make an appointment, and so on. This information would be useful for further exploring, for example, the engagement of standard requesters with the online forms typically used by PPAU. One last limitation of our study is that the standard requesters group is larger than the online requester group. Ideally, these groups would have been more comparable in size.

As far as the utility of our media campaign and form for attracting potential clients to the clinics to receive care, we found mixed results. Approximately 30% (n = 180) never showed up to clinic. This percentage is the same as PPAU’s no-show percentage; thus, our form did not improve no-show rates. However, an additional 16% of clients who eventually did show to an appointment missed their first scheduled appointment. Thus, we would suggest that contraceptive initiatives adopting an online form similar to ours might consider double booking clients initially.

Regarding the importance of our study to the general health communication literature, we reaffirm previous research arguing that media strategies can be useful for getting sexual health information across to certain groups of clients [11]. We also confirm that the use of media seems to reach those in younger cohorts especially, although there can be downsides to this. For instance, in research related to sexual health and other health concerns, it may lead to younger adults being over-represented in samples. Our study also raises questions about the significance of how media messages aimed at recruitment are constructed, since we observed fewer Hispanic participants in Period 3.

Overall, our study provides a much-needed assessment of how an advertising campaign might influence the demographics of clients who participate in contraceptive initiatives. Our media campaign also successfully attracted new clients who had never received care at a PPAU clinic before. Our findings show that while campaigns are a useful way to spread information about contraceptive initiatives and studies, care must be taken during the design stage to ensure that the advertising reaches the appropriate audiences and that design strategies are consistent with an initiative’s goals.

## Conclusion

Online media campaigns can be useful for connecting clients with advertised contraceptive methods and initiatives. However, depending on design strategy, the use of media campaigns might shift the demographics and characteristics of clients who participate in contraceptive initiatives. Our research suggests that media advertising campaigns can be useful to contraceptive initiatives, though researchers must be aware that advertising strategies may impact both participant demographics and outcomes in contraceptive initiative studies.

## Supplementary Information

**Additional file 1.** Enrollment Survey.

## Data Availability

The datasets used and/or analyzed during the current study are available from the corresponding author on reasonable request.

## Abbreviations

EHR: Electronic health records
HER Salt Lake: HER Salt Lake Contraceptive Initiative
IUD: Intrauterine device
LARC: Long-acting reversible contraception
PPAU: Planned Parenthood Association of Utah
RRR: Relative Risk Ratio
