# Supplementary material for: The HER Salt Lake media campaign: comparing characteristics and outcomes of clients who make appointments online versus standard scheduling
Source: BMC Womens Health. 2021 Mar 23;21:121. doi: 10.1186/s12905-021-01256-x (PMC7986020; doi:10.1186/s12905-021-01256-x)
Supplement: Supplementary file 1 — Additional file 1. Enrollment Survey. [file 12905_2021_1256_MOESM1_ESM.pdf]

Clinic Intake Form

This form is to be completed by clinic assistant

Intake Staff Name (First Initial Last Name) ______________________
(ex. J Sanders)

Today's Date ______________________

Language
- ◯ English
- ◯ Spanish

Date of Birth ______________________

Patient Record Number (PRN) ______________________

Enrollment Site
- ◯ South Main Clinic
- ◯ Community Health Center
- ◯ Planned Parenthood
- ◯ Other

If other, please describe ______________________

Planned Parenthood clinic
- ◯ Metro Clinic
- ◯ Salt Lake Clinic
- ◯ West Valley City Clinic
- ◯ South Jordan Clinic

What was the primary method of contraception asked for today?	◯ Contraceptive Implant (Nexplanon)<br>◯ Copper IUD (Paragard)<br>◯ Hormonal IUD (Mirena)<br>◯ Hormonal IUD (Liletta)<br>◯ Other hormonal IUD (Skyla)<br>◯ Injection (Depo-Provera)<br>◯ Combined Oral contraceptive<br>◯ Progestin Only Pill (Minipill)<br>◯ Contraceptive patch (Xulane)<br>◯ Vaginal ring (NuvaRing)<br>◯ Male condom<br>◯ Female condom<br>◯ Cervical cap or sponge<br>◯ Spermicide<br>◯ Diaphragm<br>◯ Fertility Awareness Method / Natural Family Planning / Rhythm Method<br>◯ Withdrawal<br>◯ Levonorgestrel Emergency Contraception (Plan B/Next Choice)<br>◯ Ulipristal EC Pill (Ella)<br>◯ Other<br>◯ None<br>(After contraceptive conversation)
Please describe	______________________________
Is patient using insurance today?	◯ No<br>◯ Yes<br>◯ Unsure
First Name	______________________________
Last Name	______________________________
Street, City, State, ZIP	_____________________________________
Phone number	______________________________<br>(Include Area Code)
Is this a cell phone?	◯ Yes<br>◯ No
Is it ok to text this number?	◯ Yes<br>◯ No
Is it ok to leave a message?	◯ Yes<br>◯ No
was the phone number confirmed to be a working number in clinic today?	◯ Yes<br>◯ No

E-mail	______________________ (Please confirm with patient)
Secondary E-mail	______________________
Best way to contact participant	☐ Phone call<br>☐ Text<br>☐ Email<br>☐ Other
Other, please specify:	______________________

Alternate Contact Information

Contact #1 name	______________________
Contact #1 phone number	______________________
Is it ok to leave a message?	◯ Yes<br>◯ No
Is it ok to text this number?	◯ Yes<br>◯ No
Contact #1 email address	______________________
Contact #2 name	______________________
Contact #2 phone number	______________________
Is it ok to leave a message?	◯ Yes<br>◯ No
Is it ok to text this number?	◯ Yes<br>◯ No
Contact #2 email address	______________________

Now we want to gather some info on the patients menstrual period. Please prompt participant "If you use a period tracker you may want to use that to answer the following questions."

What was the first day of your last period?

____
(Please estimate if you are not sure (if cannot provide guess please enter 9-9-9999))

How long was your previous cycle? (Cycles are from the start of one period to the start of your next period?) Typically 26-35 days

____
(Take best guess...if really unsure put 999)

On average, how many days does your period last? (That is, how many days of bleeding do you have each cycle)?

- ◯ I don't get a period
- ◯ 1-2
- ◯ 3-4
- ◯ 5-7
- ◯ 8 or more
- ◯ It varies a lot
- ◯ I prefer not to answer

In the next section, we ask questions about unprotected sex or contraceptive failures you may have had recently. Remember that your answers are completely confidential, and your honest answers really help us with our research.

Have you had sex in the last 2 weeks where you did not use a method to prevent pregnancy or used a method where you were worried it did not work (ie. condom broke, missed pill)

- ◯ No
- ◯ Yes
- ◯ I don't know
- ◯ I prefer not to answer

Please use a calendar to answer these questions:

	Check here if you had sex on that day with no method of birth control	Check here if you had sex on that day and used a method where you were worried you might get pregnant. (for example: broken condom, missed birth control pills, etc.)
1 Day ago	☐	☐
2 Days ago	☐	☐
3 Days ago	☐	☐
4 Days ago	☐	☐
5 Days ago	☐	☐
6 Days ago	☐	☐
7 Days ago	☐	☐
8 Days ago	☐	☐
9 Days ago	☐	☐
10 Days ago	☐	☐
11 Days ago	☐	☐
12 Days ago	☐	☐

13 Days ago	☐	☐
14 Days ago	☐	☐
More than 14 days ago	☐	☐

When was your most recent episode of sex when you either did not use a method or were worried about method failure?
- ◯ Within 12 hours
- ◯ Within 24 hours
- ◯ Within 48 hours
- ◯ Within 72 hours
- ◯ Within 120 hours
- ◯ Beyond 5 days

To be completed after clinic visit....

Result of urine pregnancy test done in clinic today
- ◯ Not done
- ◯ Negative
- ◯ Positive

Was IUD or implant insertion successful?
- ◯ Failed insertion
- ◯ Successful insertion

What method(s) of contraception did the patient actually leave the clinic with?
- ☐ Contraceptive Implant (Nexplanon)
- ☐ Copper IUD (Paragard)
- ☐ Hormonal IUD (Mirena)
- ☐ Hormonal IUD (Liletta)
- ☐ Other hormonal IUD (Skyla)
- ☐ Injection (Depo-Provera)
- ☐ Combined Oral contraceptive
- ☐ Progestin Only Pill (Minipill)
- ☐ Contraceptive patch (Xulane)
- ☐ Vaginal ring (NuvaRing)
- ☐ Male condom
- ☐ Female condom
- ☐ Cervical cap or sponge
- ☐ Spermicide
- ☐ Diaphragm
- ☐ Fertility Awareness Method / Natural Family Planning / Rhythm Method
- ☐ Withdrawal
- ☐ Levonorgestrel Emergency Contraception (Plan B/Next Choice)
- ☐ Ulipristal EC Pill (Ella)
- ☐ Other
- ☐ None; patient left clinic without any contraceptive method

Did the patient receive the method they desired today?
- ◯ Yes
- ◯ No

Is the method the participant left with an IUD or Implant?
- ◯ Yes
- ◯ No

Are you waiting on a sign-off on a prescription?
- ◯ Yes
- ◯ No

Is the patient scheduled to receive an IUD or implant?
- ◯ Yes
- ◯ No

What date is the patient scheduled to come back and receive their device? __________________________________

Phase/Method Switch

This form is to be completed by clinic assistant

Clinic Staff Name (First Initial Last Name)	________________ (ex. J Sanders)
Today's Date	________________
Switch Clinic Site	◯ South Main Clinic<br>◯ Community Health Center<br>◯ Planned Parenthood<br>◯ Other
If other, please describe	________________
Planned Parenthood clinic	◯ Metro Clinic<br>◯ Salt Lake Clinic<br>◯ West Valley City Clinic<br>◯ South Jordan Clinic
Phone number	________________ (Include Area Code)
E-mail	________________ (Please confirm with patient)
Is patient current on surveys?	◯ Yes<br>◯ No
What is the main reasons you came to the clinic today?	☐ Annual check-up and/or well woman exam, including pap smear<br>☐ To get birth control or change methods<br>☐ To get emergency contraception (the morning after pill)<br>☐ Pregnancy testing<br>☐ STI/STD testing<br>☐ Abortion services<br>☐ To get free or low cost birth control<br>☐ To participate in this study<br>☐ I prefer not to answer<br>☐ Other<br>(select all that apply)
If "other," please describe your reason for coming to the clinic today.	________________

How did you hear about the services that this clinic provides?	☐ Family or friend<br>☐ Partner, Boyfriend/Girlfriend, Spouse<br>☐ School class or school teacher<br>☐ Health care provider or other clinic<br>☐ Facebook/Twitter or other social media<br>☐ The website www.Bedsider.org<br>☐ Other website<br>☐ Television, radio, or newspaper<br>☐ I have been to the clinic before<br>☐ Text message sent directly to me<br>☐ I don't know<br>☐ I prefer not answer<br>☐ Other<br>(select all that apply)
If "other," please describe how you heard about the services we provide.	__________________________________
What method did you receive when you started the study?	◯ Contraceptive Implant (Nexplanon)<br>◯ Copper IUD (Paragard)<br>◯ Hormonal IUD (Mirena)<br>◯ Hormonal IUD (Liletta)<br>◯ Other hormonal IUD (Skyla)<br>◯ Injection (Depo-Provera)<br>◯ Combined Oral contraceptive<br>◯ Progestin Only Pill (Minipill)<br>◯ Contraceptive patch (Xulane)<br>◯ Vaginal ring (NuvaRing)<br>◯ Male condom<br>◯ Female condom<br>◯ Cervical cap or sponge<br>◯ Spermicide<br>◯ Diaphragm<br>◯ Fertility Awareness Method / Natural Family Planning / Rhythm Method<br>◯ Withdrawal<br>◯ Levonorgestrel Emergency Contraception (Plan B/Next Choice)<br>◯ Ulipristal EC Pill (Ella)<br>◯ Other<br>◯ None
When did you stop using the method that you received at the beginning of the study?	__________________________________<br>(Please estimate if you are not sure)

What are the reasons you are no longer using the method you received at the start of the study?	☐ Bleeding issues (spotting, irregular, ect)<br>☐ Excessive bleeding<br>☐ Cramping<br>☐ Pain<br>☐ Breast symptoms<br>☐ Weight gain<br>☐ Weight loss<br>☐ Moodiness or depression<br>☐ Bloating<br>☐ Skin problems<br>☐ Pain during intercourse<br>☐ Partner complaint<br>☐ IUD fell out/ expelled<br>☐ I wanted to get pregnant<br>☐ I had a positive pregnancy test<br>☐ Just want a different method of birth control<br>☐ Other<br>(Check all that apply)
If other, please specify:	__________________________________
What was the (new) primary method of contraception asked for today?	○ Contraceptive Implant (Nexplanon)<br>○ Copper IUD (Paragard)<br>○ Hormonal IUD (Mirena)<br>○ Hormonal IUD (Liletta)<br>○ Other hormonal IUD (Skyla)<br>○ Injection (Depo-Provera)<br>○ Combined Oral contraceptive<br>○ Progestin Only Pill (Minipill)<br>○ Contraceptive patch (Xulane)<br>○ Vaginal ring (NuvaRing)<br>○ Male condom<br>○ Female condom<br>○ Cervical cap or sponge<br>○ Spermicide<br>○ Diaphragm<br>○ Fertility Awareness Method / Natural Family Planning / Rhythm Method<br>○ Withdrawal<br>○ Levonorgestrel Emergency Contraception (Plan B/Next Choice)<br>○ Ulipristal EC Pill (Ella)<br>○ Other<br>○ None<br>(After contraceptive conversation)
Please describe	__________________________________
Is patient using insurance today?	○ No<br>○ Yes<br>○ Unsure

In the next section, we ask questions about unprotected sex or contraceptive failures you may have had recently. Remember that your answers are completely confidential, and your honest answers really help us with our research.

What was the first day of your last period?

____
(Please estimate if you are not sure (if cannot provide guess please enter 9-9-9999))

Have you had sex in the last 2 weeks where you did not use a method to prevent pregnancy or used a method where you were worried it did not work (ie. condom broke, missed pill)

- ◯ No
- ◯ Yes
- ◯ I don't know
- ◯ I prefer not to answer

Please use a calendar to answer these questions:

	Check here if you had sex on that day with no method of birth control	Check here if you had sex on that day and used a method where you were worried you might get pregnant. (for example: broken condom, missed birth control pills, etc.)
1 Day ago	☐	☐
2 Days ago	☐	☐
3 Days ago	☐	☐
4 Days ago	☐	☐
5 Days ago	☐	☐
6 Days ago	☐	☐
7 Days ago	☐	☐
8 Days ago	☐	☐
9 Days ago	☐	☐
10 Days ago	☐	☐
11 Days ago	☐	☐
12 Days ago	☐	☐
13 Days ago	☐	☐
14 Days ago	☐	☐
More than 14 days ago	☐	☐

When was your most recent episode of sex when you either did not use a method or were worried about method failure?

- ◯ Within 12 hours
- ◯ Within 24 hours
- ◯ Within 48 hours
- ◯ Within 72 hours
- ◯ Within 120 hours
- ◯ Beyond 5 days

To be completed after clinic visit....

Result of urine pregnancy test done in clinic today	◯ Not done<br>◯ Negative<br>◯ Positive
Was IUD or implant insertion successful?	◯ Failed insertion<br>◯ Successful insertion
What method(s) of contraception did the patient actually leave the clinic with?	☐ Contraceptive Implant (Nexplanon)<br>☐ Copper IUD (Paragard)<br>☐ Hormonal IUD (Mirena)<br>☐ Hormonal IUD (Liletta)<br>☐ Other hormonal IUD (Skyla)<br>☐ Injection (Depo-Provera)<br>☐ Combined Oral contraceptive<br>☐ Progestin Only Pill (Minipill)<br>☐ Contraceptive patch (Xulane)<br>☐ Vaginal ring (NuvaRing)<br>☐ Male condom<br>☐ Female condom<br>☐ Cervical cap or sponge<br>☐ Spermicide<br>☐ Diaphragm<br>☐ Fertility Awareness Method / Natural Family Planning / Rhythm Method<br>☐ Withdrawal<br>☐ Levonorgestrel Emergency Contraception (Plan B/Next Choice)<br>☐ Ulipristal EC Pill (Ella)<br>☐ Other<br>☐ None; patient left clinic without any contraceptive method
Did the patient receive the method they desired today?	◯ Yes<br>◯ No
Is the method the participant left with an IUD or Implant?	◯ Yes<br>◯ No
Are you waiting on a sign-off on a prescription?	◯ Yes<br>◯ No
Is the patient scheduled to receive an IUD or implant?	◯ Yes<br>◯ No
What date is the patient scheduled to come back and receive their device?	______________________
Additional Comments	______________________

Final Study Status

STUDY COMPLETION

Current Study Status:
- ◯ Ongoing
- ◯ Completed study
- ◯ Lost to follow-up
- ◯ Withdrawn form study
- ◯ Investigator decision
- ◯ Other

Other, please specify: ______________________________

Date completed study ______________________________

Lost to follow-up notes ______________________________

Date withdrawn from study ______________________________

Reason for withdrawal: ______________________________

Baseline Method Outcome
- ◯ Continuing Method
- ◯ Switched Method
- ◯ Discontinued all birth control
- ◯ Pregnancy
- ◯ Lost-to-follow-up

Date of method outcome ______________________________

Last Survey Completed
- ◯ enrollment
- ◯ 2-week
- ◯ 4-week
- ◯ 3-month
- ◯ 6-month
- ◯ 9-month
- ◯ 12-month
- ◯ 18-month
- ◯ 24-month
- ◯ 30-month
- ◯ 36-month

Enrollment Survey

Welcome! Although 99% of women use birth control at some point in their lives, we still have lots to learn. This study will help us learn more about where people get information on birth control, as well as more about the relationship between birth control and people's sexual experiences. Findings could help improve the quality of reproductive health care.

We estimate that this survey will take you 15-30 minutes to complete. Once you complete the survey, you will receive a $20 gift card as a thank you for your time.

Do your best to answer each question, and remember that all information is completely confidential.

Thank you warmly for your participation.

We want to ask you about your visit to the clinic today.

What is the main reasons you came to the clinic today?

- ☐ Annual check-up and/or well woman exam, including pap smear
- ☐ To get birth control or change methods
- ☐ To get emergency contraception (the morning after pill)
- ☐ Pregnancy testing
- ☐ STI/STD testing
- ☐ Abortion services
- ☐ To get free or low cost birth control
- ☐ To participate in this study
- ☐ I prefer not to answer
- ☐ Other

(select all that apply)

If "other," please describe your reason for coming to the clinic today.

____

How did you hear about the services that this clinic provides?

- ☐ Family or friend
- ☐ Partner, Boyfriend/Girlfriend, Spouse
- ☐ School class or school teacher
- ☐ Health care provider or other clinic
- ☐ Facebook/Twitter or other social media
- ☐ Planned Parenthood advertising (ProUtah, etc.)
- ☐ The website www.Bedsider.org
- ☐ Other website
- ☐ Television, radio, or newspaper
- ☐ I have been to the clinic before
- ☐ Text message sent directly to me
- ☐ www.hersaltlake.org
- ☐ Other
- ☐ I don't know
- ☐ I prefer not answer

(select all that apply)

If "other," please describe how you heard about the services we provide.

____

Have you ever visited the website www.hersaltlake.org?

- ○ No
- ○ Yes
- ○ I don't know
- ○ I prefer not to answer

Where did you learn about www.hersaltlake.org?

- ☐ Word of mouth (family or friend)
- ☐ Online Ad Banner
- ☐ Facebook
- ☐ Instagram
- ☐ Twitter
- ☐ Newspaper, Radio,
- ☐ Other

Have you ever visited the website www.proutah.org?

- ○ No
- ○ Yes
- ○ I don't know
- ○ I prefer not to answer

Where did you see Planned Parenthood advertising or learn about proutah.org?	☐ Online publications<br>☐ Public transportation<br>☐ Billboards<br>☐ Radio<br>☐ Social Media<br>☐ Other
Have you ever visited the website www.bedsider.org?	◯ No<br>◯ Yes<br>◯ I don't know<br>◯ I prefer not to answer
If you have visited the website www.bedsider.org, how did you learn about it?	☐ Family or friend<br>☐ Partner, Boyfriend/Girlfriend, Spouse<br>☐ School class or school teacher<br>☐ Health care provider or other clinic<br>☐ Facebook/Twitter or other social media<br>☐ The website www.Bedsider.org<br>☐ Other website<br>☐ Message sent directly to my phone<br>☐ Television, radio, or newspaper<br>☐ Other<br>☐ I have been to the clinic before<br>☐ I don't know<br>☐ I prefer not answer<br>(select all that apply)
If other, please specify:	______________________________

Participant Background

First, please tell us a little bit more about yourself. Your answers will NOT impact participation in the study, clinical care, or any services you receive.

Were you born in the United States?	◯ No<br>◯ Yes<br>◯ I prefer not to answer
In what country were you born?	__________________________________
What year did you move to the United States?	__________________________________
We understand that not everyone who needs or wants birth control identifies as a woman.<br><br>Please select the word(s) you use to describe yourself, or select self-describe and you can write in the word(s) you use.	☐ Woman<br>☐ Man<br>☐ Non-binary<br>☐ Transgender<br>☐ Prefer to self-describe<br>(check all that apply)
Please describe yourself.	__________________________________
Which of the following best describes your ethnicity/race?	☐ White<br>☐ Hispanic or Latina<br>☐ Asian<br>☐ Native Hawaiian or Pacific Islander<br>☐ American Indian or Alaska Native<br>☐ African American or Black<br>☐ Other<br>☐ I don't know<br>☐ I prefer not to answer
If other please describe.	__________________________________
Which of the following best describes your religious identity?	◯ Not Religious<br>◯ Christian (Protestant, Evangelical, Mainline, etc)<br>◯ Catholic<br>◯ Mormon<br>◯ Jewish<br>◯ Muslim<br>◯ Other<br>◯ Don't know or prefer not to answer
Please describe your religious identity.	__________________________________

Which of the following best describes your current relationship situation?	◯ Married<br>◯ Not married, but living together or in a committed relationship<br>◯ Actively dating, but NOT in a committed relationship<br>◯ Divorced/Separated<br>◯ Single, not in a relationship<br>◯ Widowed<br>◯ Other<br>◯ I prefer not to answer
Other, please describe	__________________________________
Please choose the description that best fits how you think of yourself.	◯ Exclusively Heterosexual (Straight)<br>◯ Mostly Heterosexual<br>◯ Bisexual (Attracted to both Men and Women)<br>◯ Mostly Gay/Lesbian<br>◯ Exclusively Homosexual (Gay/Lesbian)<br>◯ Not sexually attracted to either males or females<br>◯ Other<br>◯ I don't know<br>◯ I prefer not to answer
In the last 12 months have you had sex with...	◯ Not currently sexually active<br>◯ Males only<br>◯ Both males and females<br>◯ Females only<br>◯ I prefer not to answer
Considering all types of sexual activity, how many female partners have you EVER had sex with? (If you can't remember exactly, just make your best guess.)	__________________________________
Considering all types of sexual activity, with how many female partners have you had sex in the past 12 months, even if only one time?	__________________________________
What type of medical insurance do you currently have?	◯ None<br>◯ Medicaid<br>◯ Insurance through your job or that you buy on your own<br>◯ Student health insurance<br>◯ Parent's insurance<br>◯ Military or VA (Champus, ChampVA, Tricare)<br>◯ Disability or Medicare<br>◯ I don't know<br>◯ I prefer not to answer

The next few questions are about your current employment.

What best describes your current employment status?

- ☐ Unemployed
- ☐ Working full-time (at least 30 hrs/wk)
- ☐ Working part-time (less than 30 hrs/wk)
- ☐ Disabled, sick leave, family leave
- ☐ Retired
- ☐ Homemaker
- ☐ Student
- ☐ Other
- ☐ I prefer not to answer

(select all that apply)

If other employment, please describe.

____

How many hours do you work each week?

____
(Please guess if you are not sure)

What is your hourly wage?

____
(Example 7.25 if you make $7.25 per hour....Please estimate if you are not sure)

What kind of work do you do?

____

Are you currently looking for work, additional work, or different work?

- ◯ No
- ◯ Yes
- ◯ Don't know
- ◯ I prefer not to answer this question

The next few questions are about your education and education level of your parents.

What best describes the highest level of education you have COMPLETED SO FAR?

- ◯ 11th grade or less
- ◯ 12th grade (completed high school or GED)
- ◯ Vocational/technical training
- ◯ Associate degree or some college
- ◯ 4-year college degree (BA/BS)
- ◯ Any graduate or professional education (any time in a Masters, JD, PhD, MD, etc. program)
- ◯ I don't know
- ◯ I prefer not to answer

Are you currently in school, either full-time or part-time?

- ◯ I'm not currently in school
- ◯ Part-time
- ◯ Full-time
- ◯ I prefer not to answer this question

What best describes your PLANS for the highest level of education in the future?

- ◯ None, I am done with school and do not have plans to go back
- ◯ I plan to finish high school or GED
- ◯ Get vocational/technical training
- ◯ Get an associate degree
- ◯ Get a 4-year college degree (BA/BS)
- ◯ Get graduate or professional education (Masters, JD PhD, MD)
- ◯ I don't know
- ◯ I prefer not to answer

What date do you expect to graduate?

____

What is the highest level of education your mother (or female guardian) completed?

- ◯ Less than High School
- ◯ High School degree or GED
- ◯ Vocational/technical training
- ◯ Associate degree or some college
- ◯ 4-year college degree (BA/BS)
- ◯ Any graduate or professional education (any time in a Masters, JD, PhD, MD, etc.)
- ◯ I don't know
- ◯ I prefer not to answer

What is the highest level of education your father (or male guardian) completed?

- ◯ Less than High School
- ◯ High School degree or GED
- ◯ Vocational/technical training
- ◯ Associate degree or some college
- ◯ 4-year college degree (BA/BS)
- ◯ Any graduate or professional education (any time in a Masters, JD, PhD, MD, etc.
- ◯ I don't know
- ◯ I prefer not to answer

Thank you for you answers. Just a few more questions about your financial situation. Again, these will not impact your participation or clinical care.

Please select the range of your yearly household income?

- ◯ Less than $10,000
- ◯ $10,000-$19,999
- ◯ $20,000-$29,999
- ◯ $30,000-$39,999
- ◯ $40,000-$49,999
- ◯ $50,000-$59,999
- ◯ $60,000-$69,999
- ◯ $70,000-$79,999
- ◯ $80,000 or more
- ◯ I don't know
- ◯ I prefer not to answer this question

What is your best estimate of your yearly household income?

____
(8,500 if your income is $8,500 per year)

How many people are in your household?

- ◯ 1
- ◯ 2
- ◯ 3
- ◯ 4
- ◯ 5
- ◯ 6
- ◯ 7
- ◯ 8
- ◯ 9
- ◯ 10
- ◯ 11
- ◯ 12
- ◯ 13
- ◯ 14
- ◯ 15+

(including you)

How many children under the age of 18 do you have living with you?

____

Please check all of the following that have been a source of income in the last month:

- ☐ Self
- ☐ Spouse or Partner
- ☐ Other family member
- ☐ Government assistance
- ☐ Other
- ☐ I prefer not to answer

(select all that apply)

What other sources of income did you receive in the past month?

____

How much money did you make last month?	◯ None<br>◯ $1-$400<br>◯ $401-$800<br>◯ $801-$1,200<br>◯ $1,201-$1,600<br>◯ $1,601-$2,000<br>◯ $2,001-$2,400<br>◯ $2,401-$2,800<br>◯ More than $2,800<br>◯ Don't know<br>◯ I prefer not to answer
How much money did your partner or spouse make last month?	◯ None<br>◯ $1-$400<br>◯ $401-$800<br>◯ $801-$1,200<br>◯ $1,201-$1,600<br>◯ $1,601-$2,000<br>◯ $2,001-$2,400<br>◯ $2,401-$2,800<br>◯ More than $2,800<br>◯ Don't know<br>◯ I prefer not to answer
How much money did your other family members contribute to your household income?	◯ None<br>◯ $1-$400<br>◯ $401-$800<br>◯ $801-$1,200<br>◯ $1,201-$1,600<br>◯ $1,601-$2,000<br>◯ $2,001-$2,400<br>◯ $2,401-$2,800<br>◯ More than $2,800<br>◯ Don't know<br>◯ I prefer not to answer
How much money did government assistance contribute to your household income last month?	◯ None<br>◯ $1-$400<br>◯ $401-$800<br>◯ $801-$1,200<br>◯ $1,201-$1,600<br>◯ $1,601-$2,000<br>◯ $2,001-$2,400<br>◯ $2,401-$2,800<br>◯ More than $2,800<br>◯ Don't know<br>◯ I prefer not to answer
How much money did you receive from other sources last month?	◯ None<br>◯ $1-$400<br>◯ $401-$800<br>◯ $801-$1,200<br>◯ $1,201-$1,600<br>◯ $1,601-$2,000<br>◯ $2,001-$2,400<br>◯ $2,401-$2,800<br>◯ More than $2,800<br>◯ Don't know<br>◯ I prefer not to answer<br>(this includes child support)

Were you supposed to receive any child support in the last 4 weeks?	◯ No<br>◯ Yes<br>◯ I prefer not to answer
How much child support were you supposed to receive?	__________________________________
Did you receive any child support in the last 4 weeks?	◯ No<br>◯ Yes<br>◯ I prefer not to answer this question
How much child support did you receive in the last 4 weeks?	__________________________________
Which of the following best describes your current housing situation?	◯ Homeless<br>◯ Shelter<br>◯ Mobile home<br>◯ Apartment<br>◯ Single-family house<br>◯ Other<br>◯ I prefer not to answer this question
Describe type of housing	__________________________________

We will now ask a few questions about public assistance that you may receive:

Do you currently receive:

	No	Yes	Prefer not to answer
Food stamps	○	○	○
WIC (Women, Infants and Children)	○	○	○
Welfare	○	○	○
Unemployment Benefits	○	○	○

Just a few more questions about your economic situation.

During the past 12 months, have you had trouble paying for the following:

	No	Yes	Prefer not to answer
Transportation	◯	◯	◯
Housing	◯	◯	◯
Medical care or medications	◯	◯	◯
Food	◯	◯	◯

During the past month, how often would you say you had enough money to meet your basic living needs such as food, housing and transportation?

◯ All the time
◯ Most of the time
◯ Some of the time
◯ Rarely
◯ Never
◯ Don't know
◯ I prefer not to answer

Now we want to ask you some questions about your pregnancy history and outcomes.

Have you ever been pregnant before?
- ◯ No
- ◯ Yes
- ◯ Prefer not to answer

How many times in your life have you been pregnant
- ◯ 0
- ◯ 1
- ◯ 2
- ◯ 3
- ◯ 4
- ◯ 5
- ◯ 6
- ◯ 7
- ◯ 8
- ◯ 9
- ◯ 10+

How many times in your life have you had a live birth?
- ◯ 0
- ◯ 1
- ◯ 2
- ◯ 3
- ◯ 4
- ◯ 5
- ◯ 6
- ◯ 7
- ◯ 8
- ◯ 9
- ◯ 10+

How many times in your life have you had a miscarriage?
- ◯ 0
- ◯ 1
- ◯ 2
- ◯ 3
- ◯ 4
- ◯ 5
- ◯ 6
- ◯ 7
- ◯ 8
- ◯ 9
- ◯ 10+

How many times in your life have you had an abortion?
- ◯ 0
- ◯ 1
- ◯ 2
- ◯ 3
- ◯ 4
- ◯ 5
- ◯ 6
- ◯ 7
- ◯ 8
- ◯ 9
- ◯ 10+

How many times in your life have you had a stillbirth?

◯ 0
◯ 1
◯ 2
◯ 3
◯ 4
◯ 5
◯ 6
◯ 7
◯ 8
◯ 9
◯ 10+

How many times in your life have you had an ectopic (tubal) pregnancy?

◯ 0
◯ 1
◯ 2
◯ 3
◯ 4
◯ 5
◯ 6
◯ 7
◯ 8
◯ 9
◯ 10+

How many times in your life have you placed a child for adoption?

◯ 0
◯ 1
◯ 2
◯ 3
◯ 4
◯ 5
◯ 6
◯ 7
◯ 8
◯ 9
◯ 10+

How many times in your life have you been pregnant when you did not want to be?

◯ 0
◯ 1
◯ 2
◯ 3
◯ 4
◯ 5
◯ 6
◯ 7
◯ 8
◯ 9
◯ 10+

How old were you when you became pregnant for the FIRST time?

____

When did your MOST RECENT pregnancy end? If you can't remember the exact date, please make your best guess.

____

How did your most recent pregnancy end?

- ◯ Miscarriage
- ◯ Abortion
- ◯ Preterm live birth (less than 37 weeks)
- ◯ Term live birth (37 weeks or more)
- ◯ Ectopic (tubal)
- ◯ Stillbirth (20 weeks or more)
- ◯ Prefer not to answer

Now we would like to ask you about your plans for future children, ideal timing, and feelings surrounding pregnancy.

Do you think you would like to have children (or more children) in the future?

- ◯ No
- ◯ Yes
- ◯ I don't know

What are your future pregnancy plans?

- ◯ I do not plan on getting pregnant at any time in the future.
- ◯ I would like to get pregnant in the next year.
- ◯ I would like to get pregnant in the next 2-5 years (but not in the next year).
- ◯ I would like to get pregnant in the next 5-10 years but not before then.
- ◯ I am uncertain of if or when I would like to become pregnant.
- ◯ Other

What are your pregnancy plans?

____

How important is it to you to not get pregnant until you are ready?

not at all important — the most important

(Place a mark on the scale above)

How important is it to you to not get pregnant now or in the future?

not at all important — the most important

(Place a mark on the scale above)

How would you feel about getting pregnant in the next month?

worst feeling you can imagine — happiest you could possibly feel

(Place a mark on the scale above)

Please tell us a bit more about how a pregnancy now or in the next few weeks would affect your life.

____

Now we want to ask you some questions about methods you have used to prevent pregnancy.

What method(s) to prevent pregnancy have you EVER used in the past?

- ☐ Contraceptive Implant (Nexplanon)
- ☐ Copper IUD (Paragard)
- ☐ Hormonal IUD (Mirena)
- ☐ Hormonal IUD (Liletta)
- ☐ Other hormonal IUD (Skyla)
- ☐ Injection (Depo-Provera)
- ☐ Combined oral contraceptive pill (The Pill)
- ☐ Progestin Only Pill (Minipill)
- ☐ Contraceptive patch (Xulane or OrthoEvra)
- ☐ Vaginal ring (NuvaRing)
- ☐ Male condom
- ☐ Female condom
- ☐ Cervical cap or sponge
- ☐ Spermicide
- ☐ Diaphragm
- ☐ Fertility Awareness Method / Natural Family Planning / Rhythm Method
- ☐ Withdrawal
- ☐ Levonorgestrel Emergency Contraception (Plan B/Next Choice)
- ☐ Ulipristal EC Pill (Ella)
- ☐ Other
- ☐ None; I've never used contraception

(select all that apply)

If other, please specify:

____

What method(s) have you used in the last 4 weeks?

This should not include the method you are receiving today.

- ☐ Contraceptive Implant (Nexplanon)
- ☐ Copper IUD (Paragard)
- ☐ Hormonal IUD (Mirena)
- ☐ Hormonal IUD (Liletta)
- ☐ Other hormonal IUD (Skyla)
- ☐ Injection (Depo-Provera)
- ☐ Combined oral contraceptive pill (The Pill)
- ☐ Progestin Only Pill (Minipill)
- ☐ Contraceptive patch (Xulane or OrthoEvra)
- ☐ Vaginal ring (NuvaRing)
- ☐ Male condom
- ☐ Female condom
- ☐ Cervical cap or sponge
- ☐ Spermicide
- ☐ Diaphragm
- ☐ Fertility Awareness Method / Natural Family Planning / Rhythm Method
- ☐ Withdrawal
- ☐ Levonorgestrel Emergency Contraception (Plan B/Next Choice)
- ☐ Ulipristal EC Pill (Ella)
- ☐ Abstinence
- ☐ Other
- ☐ None; I have not used anything to avoid pregnancy in the last 4 weeks

(select all that apply)

If other, please specify:

____

How long have you been on this method of birth control?<br><br>If you are using multiple methods answer for any hormonal method.	◯ less than 3 months<br>◯ 3 to 6 months<br>◯ 6 months to 1 year<br>◯ 1 to 2 years<br>◯ 2 to 3 years<br>◯ more than 3 years<br>◯ I don't know<br>◯ I prefer not to answer
How many years have you used this method of birth control?	__________________________________
Overall, how satisfied are you with the method you were using during the previous 4 weeks?	◯ Completely satisfied<br>◯ Somewhat satisfied<br>◯ Neither satisfied or dissatisfied<br>◯ Somewhat dissatisfied<br>◯ Completely dissatisfied<br>◯ I prefer not to answer
Overall, how confident were you that the method you have been using the past 4 weeks will prevent pregnancy?	◯ Very high confidence<br>◯ High confidence<br>◯ Moderate confidence<br>◯ Low confidence<br>◯ Very low or no confidence<br>◯ I prefer not to answer
Please rate your agreement or disagreement with the following statement: "I feel that I have control over whether or not I get pregnant."	◯ I strongly agree<br>◯ I somewhat agree<br>◯ I neither agree nor disagree<br>◯ I somewhat disagree<br>◯ I strongly disagree<br>◯ I prefer not to answer
Before today had you heard of the IUD?	◯ Yes<br>◯ No
Which best describes your level of interest in getting an IUD for birth control today?	◯ Not at all interested<br>◯ Somewhat interested<br>◯ Extremely interested
If you could have an IUD placed today for free, would you want one?	◯ No<br>◯ Yes<br>◯ Not sure
Did you want an IUD but were not able to get it today?	◯ No<br>◯ Yes<br>◯ Not sure
If yes, explain why:	__________________________________
Before today had you heard of the contraceptive implant, Nexplanon?	◯ Yes<br>◯ No
Which best describes your level of interest in getting an implant for birth control today?	◯ Not at all interested<br>◯ Somewhat interested<br>◯ Extremely interested

If you could have an implant placed today for free, would you want one?	◯ No<br>◯ Yes<br>◯ Not sure
Did you want an implant but were not able to get it today?	◯ No<br>◯ Yes<br>◯ Not sure
If yes, explain why:	__________________________________

How important are each of the following characteristics to you in deciding which birth control method to use?

	Not at all important	Slightly important	Quite important	Extremely important	I don't know/ I prefer not to answer
It doesn't contain hormones	○	○	○	○	○
It is acceptable to my partner	○	○	○	○	○
It doesn't interrupt sex	○	○	○	○	○
It doesn't reduce my libido	○	○	○	○	○
It is in line with my religious beliefs	○	○	○	○	○
It is recommended by my friend(s)	○	○	○	○	○
It is the most effective method	○	○	○	○	○
It doesn't have side effects	○	○	○	○	○
It is safe for me to use	○	○	○	○	○

Now we want to ask you a few questions about certain aspects of your health and well-being that may be related to your menstrual cycle. You may still experience these things if you don't have a period.

In the past 4 weeks, have any the following health issues been a problem for you?

	Have not had in the past 30 days	Once a month	A couple of days a month	Once a week	A couple of days a week	Everyday	Don't know or prefer not to answer
Headaches	◯	◯	◯	◯	◯	◯	◯
Bloating	◯	◯	◯	◯	◯	◯	◯
Breast Tenderness	◯	◯	◯	◯	◯	◯	◯
Moodiness or irritability	◯	◯	◯	◯	◯	◯	◯
Acne flare-up	◯	◯	◯	◯	◯	◯	◯
Cramping	◯	◯	◯	◯	◯	◯	◯
Weight Gain	◯	◯	◯	◯	◯	◯	◯
Weight Loss	◯	◯	◯	◯	◯	◯	◯
Depressed mood	◯	◯	◯	◯	◯	◯	◯
Constipation or diarrhea	◯	◯	◯	◯	◯	◯	◯

Have you sought medical care for any of these conditions?

◯ No
◯ Yes
◯ Don't know

If so, please specify which one(s): ______________________

Were you prescribed medication to treat these conditions?

◯ No
◯ Yes

Next, we want to gather information about your sexual relationships. Please remember that everything you say is confidential.

On a scale of 1 to 100, how might you rank your sex life right now?

worst possible — best possible

(Place a mark on the scale above)

In the last 4 weeks, would you say that your birth control or method to avoid pregnancy has:

- ◯ Improved my sex life a lot
- ◯ Improved my sex life a little
- ◯ Has had no effect on my sex life
- ◯ Has made my sex life a little worse
- ◯ Has made my sex life a lot worse

Briefly explain the impact your method used in the last 4 weeks to avoid pregnancy has on your sex life.

____

Have you been sexually active with a male partner in the past four weeks? This may include a variety of activities, not just vaginal intercourse.

- ◯ No
- ◯ Yes
- ◯ I prefer not to answer

How long have you been in your sexual relationship?

Note: If you have more than one partner, think about your primary/main partner when answering.

- ◯ Less than 3 months
- ◯ 3 to 6 months
- ◯ 6 months to 1 year
- ◯ 1 to 2 years
- ◯ 2 to 3 years
- ◯ More than 3 years

If you have been in a relationship with your primary sexual partner for more than 3 years, please tell us how many years it has been.

____
(years)

We will now ask you a few questions about your sexual feelings and responses during the past four weeks.

Over the past 4 weeks, how would you rate your level (degree) of sexual desire or interest?	◯ Very high<br>◯ High<br>◯ Moderate<br>◯ Low<br>◯ Very low or none at all<br>◯ Prefer not to answer
Over the past 4 weeks, how would you rate your level of sexual arousal ("turn on") during sexual activity or intercourse?	◯ No sexual activity<br>◯ Very high<br>◯ High<br>◯ Moderate<br>◯ Low<br>◯ Very low or none at all<br>◯ Prefer not to answer
Over the past 4 weeks, how often did you become lubricated ("wet") during sexual activity or intercourse?	◯ No sexual activity<br>◯ Almost always or always<br>◯ Most times (more than half the time)<br>◯ Sometimes (about half the time)<br>◯ A few times (less than half the time)<br>◯ Almost never or never<br>◯ Prefer not to answer
Over the past 4 weeks, when you had sexual stimulation or intercourse, how often did you reach orgasm (climax)?	◯ No sexual activity<br>◯ Almost always or always<br>◯ Most times (more than half the time)<br>◯ Sometimes (about half the time)<br>◯ A few times (less than half the time)<br>◯ Almost never or never<br>◯ Prefer not to answer
Over the past 4 weeks, how satisfied have you been with your overall sexual life?	◯ Very satisfied<br>◯ Moderately satisfied<br>◯ About equally satisfied and dissatisfied<br>◯ Moderately dissatisfied<br>◯ Very dissatisfied<br>◯ Prefer not to answer
Over the past 4 weeks, how often did you experience discomfort or pain during vaginal penetration?	◯ Did not attempt intercourse<br>◯ Almost always or always<br>◯ Most times (more than half the time)<br>◯ Sometimes (about half the time)<br>◯ A few times (less than half the time)<br>◯ Almost never or never<br>◯ Prefer not to answer

The following items relate to your sexual experiences. You might find a few of the items similar to items you just filled out. That's okay; just do your best to answer all the questions.

When responding to these items, please think about the last 4 weeks.

Thinking about the last month, how satisfied or dissatisfied are you with each of the following issues?

	not at all satisfied	a little satisfied	moderately satisfied	very satisfied	extremely satisfied	I prefer not to answer
The intensity of my sexual arousal	◯	◯	◯	◯	◯	◯
The quality of my orgasms	◯	◯	◯	◯	◯	◯
My letting go and surrender to sexual pleasure during sex	◯	◯	◯	◯	◯	◯
My focus and concentration during sexual activity	◯	◯	◯	◯	◯	◯
The way I sexually react to my partner	◯	◯	◯	◯	◯	◯
My body's sexual functioning	◯	◯	◯	◯	◯	◯
My emotional opening up in sex	◯	◯	◯	◯	◯	◯
My mood after sexual activity	◯	◯	◯	◯	◯	◯
The frequency of my orgasms	◯	◯	◯	◯	◯	◯
The pleasure I provide to my partner	◯	◯	◯	◯	◯	◯
The balance between what I give and receive in sex	◯	◯	◯	◯	◯	◯
My partner's emotional opening up during sex	◯	◯	◯	◯	◯	◯
My partner's initiation of sexual activity	◯	◯	◯	◯	◯	◯
My partner's ability to orgasm	◯	◯	◯	◯	◯	◯
My partner's surrender to sexual pleasure (letting go)	◯	◯	◯	◯	◯	◯
The way my partner takes care of my sexual needs	◯	◯	◯	◯	◯	◯
My partner's sexual creativity	◯	◯	◯	◯	◯	◯
My partner's sexual availability	◯	◯	◯	◯	◯	◯
The variety of my sexual activities	◯	◯	◯	◯	◯	◯
The frequency of my sexual activity	◯	◯	◯	◯	◯	◯

Do you have any concern about your sexual functioning?

◯ No
◯ Yes
◯ I don't know

Please briefly describe.

____

The last few items have to do with more general health and well-being. Please think about your experiences in the last four weeks when answering.

	All of the time	Most of the time	More than half of the time	Less than half of the time	Some of the time	At no time
I have felt cheerful and in good spirits	◯	◯	◯	◯	◯	◯
I have felt calm and relaxed	◯	◯	◯	◯	◯	◯
I have felt active and vigorous	◯	◯	◯	◯	◯	◯
I woke up feeling fresh and rested	◯	◯	◯	◯	◯	◯
My daily life has been filled with things that interest me	◯	◯	◯	◯	◯	◯

Would you be willing to have study staff contact you for future studies?

◯ Yes
◯ No

Is there anything else you would like us to know?

____

Followup Survey (1, 3, & 6 months)

Welcome back! Thank you again for participating. We would like to follow-up with you about your experiences since you enrolled in this study. Your feedback could help us improve the quality of reproductive health care.

We estimate that this survey will take you less than 10 minutes to complete. Once you complete the survey, you will receive a credit toward a $20 gift card which you will be emailed after the 6 month survey.

Do your best to answer each question, and remember that all information is completely confidential.

Thank you warmly for your participation.

Thank you very much for participating in this study.

First we want to make sure you haven't changed any of your contact information.

Has any of your contact information changed (for example, your phone number, email, or mailing address)?	◯ No<br>◯ Yes
If so, which contact info has changed? Please check all that apply.	☐ New phone number<br>☐ New email<br>☐ New address<br>(check all that apply)
Please list your new phone number.	______________________<br>(xxx-xxx-xxxx)
Please list your new email address.	______________________
Please list your new mailing address.	____________________________

We will start by asking you a few questions about your contraceptive method(s).

What method(s) to prevent pregnancy have you used in the last 4 weeks?

- ☐ Contraceptive Implant (Nexplanon)
- ☐ Copper IUD (Paragard)
- ☐ Hormonal IUD (Mirena)
- ☐ Hormonal IUD (Liletta)
- ☐ Other hormonal IUD (Skyla)
- ☐ Injection (Depo-Provera)
- ☐ Combined oral contraceptive pill (The Pill)
- ☐ Progestin Only Pill (Minipill)
- ☐ Contraceptive patch (Xulane or OrthoEvra)
- ☐ Vaginal ring (NuvaRing)
- ☐ Male condom
- ☐ Female condom
- ☐ Cervical cap or sponge
- ☐ Spermicide
- ☐ Diaphragm
- ☐ Fertility Awareness Method / Natural Family Planning / Rhythm Method
- ☐ Withdrawal
- ☐ Levonorgestrel Emergency Contraception (Plan B/Next Choice)
- ☐ Ulipristal EC Pill (Ella)
- ☐ Other
- ☐ None; I am not using any contraceptive method

(select all that apply)

If other, please specify:

____

Are you still using the same method of contraception that you received at the beginning of this study?

- ◯ Yes
- ◯ No

In the last 4 weeks, have you checked to make sure your IUD or implant is still in place?

- ◯ No, I have not
- ◯ Yes, I felt my strings (or rod in my arm) myself and confirmed its still there
- ◯ Yes, I had a provider check
- ◯ I tried but was not able to feel anything

How long do you plan on using the birth control method that you are currently using?

- ◯ Less than 1 year
- ◯ More than 1 year but less than 2 years
- ◯ More than 2 years but less than 3 years
- ◯ More than 3 years but less than 5 years
- ◯ More than 5 years but less than 10 years
- ◯ More than 10 years
- ◯ Unsure
- ◯ I prefer not to answer this question

What are the reasons you are no longer using the method you received at the start of the study?	☐ Bleeding issues (spotting, irregular, ect)<br>☐ Excessive bleeding<br>☐ Cramping<br>☐ Pain<br>☐ Breast symptoms<br>☐ Weight gain<br>☐ Weight loss<br>☐ Moodiness or depression<br>☐ Bloating<br>☐ Skin problems<br>☐ Loss of libido/sexual interest<br>☐ Pain during intercourse<br>☐ Partner complaint<br>☐ IUD fell out/ expelled<br>☐ I wanted to get pregnant<br>☐ I had a positive pregnancy test<br>☐ Other<br>(Check all that apply)
If other, please specify:	______________________________
When did you stop using the method that you received at the beginning of the study?	______________________________<br>(Please estimate if you are not sure)
When did you start using your new method?	______________________________<br>(please make you best guess)
Which of the following best describes your vaginal bleeding in the last 4 weeks?	◯ I've had no vaginal bleeding<br>◯ I've had less bleeding than before<br>◯ I've had no change from before<br>◯ I've had more bleeding than before

The next questions are about any pregnancy or health issues that may be related to your contraceptive method(s) since you entered the study.

In the last four weeks, have you taken a pregnancy test?	◯ No<br>◯ Yes, I took a test at home<br>◯ Yes, I took a test in a clinic<br>◯ I don't know
What were the results of your pregnancy test?	◯ Negative<br>◯ Positive<br>◯ I don't know
What was the date of your positive pregnancy test?	______________________
When you got pregnant, were you trying to get pregnant?	◯ Yes<br>◯ No
If you have had a positive pregnancy test, please indicate the outcome of the pregnancy.	◯ I had or am planning to have an abortion<br>◯ I had a miscarriage<br>◯ I had an ectopic pregnancy<br>◯ I am planning on continuing the pregnancy and keeping the baby<br>◯ I am planning on continuing the pregnancy and placing the baby up for adoption<br>◯ I am unsure of what I am going to do
What was the date when your pregnancy ended, regardless of the outcome? If you are still pregnant and continuing the pregnancy please put your estimated due date?	______________________
To ensure your safety, if you had a pregnancy, we will follow-up on the care you received. Please provide the name of the clinic or hospital where you were seen.	____________________________
Since enrolling in the study, have you been hospitalized for any illness or injury?	◯ Yes<br>◯ No
Please provide the date:	______________________
Please describe what happened:	____________________________
Have you seen a medical provider for an issue that you thought might be related to your contraception?	◯ No<br>◯ Yes
Please provide the date:	______________________
Please describe:	____________________________

Now we want to ask you some questions about contraception. Think about the method(s) you have been using in the last 4 weeks.

Overall, how satisfied are you with the method(s) you were using during the last 4 weeks?	◯ Completely satisfied<br>◯ Somewhat satisfied<br>◯ Neither satisfied or dissatisfied<br>◯ Somewhat dissatisfied<br>◯ Completely dissatisfied<br>◯ I prefer not to answer
Overall, how confident were you that the method you have been using the last 4 weeks will prevent pregnancy?	◯ Very high confidence<br>◯ High confidence<br>◯ Moderate confidence<br>◯ Low confidence<br>◯ Very low or no confidence<br>◯ I prefer not to answer
Please rate your agreement or disagreement with the following statement: "I feel that I have control over whether or not I get pregnant."	◯ I strongly agree<br>◯ I somewhat agree<br>◯ I neither agree nor disagree<br>◯ I somewhat disagree<br>◯ I strongly disagree<br>◯ I prefer not to answer

Now we want to ask you a few questions about certain aspects of your health and well-being that may be related to your menstrual cycle. You may still experience these things even if you don't have a period.

In the last 4 weeks, have any the following health issues been a problem for you?

	Have not had in the past 30 days	Once a month	A couple of days a month	Once a week	A couple of days a week	Everyday	Don't know
Headaches	○	○	○	○	○	○	○
Bloating	○	○	○	○	○	○	○
Breast tenderness	○	○	○	○	○	○	○
Moodiness or irritability	○	○	○	○	○	○	○
Acne flare-up	○	○	○	○	○	○	○
Cramping	○	○	○	○	○	○	○
Weight gain	○	○	○	○	○	○	○
Weight loss	○	○	○	○	○	○	○
Depressed mood	○	○	○	○	○	○	○
Constipation or diarrhea	○	○	○	○	○	○	○

Have you sought medical care for any of these conditions?

○ Yes
○ No
○ Don't know

If so, please specify which one(s): ______________________

Were you prescribed medication to treat these conditions?

○ Yes
○ No

Next, we want to gather information about your sexual relationships. Please remember that everything you say is confidential.

Please choose the description that best fits how you think about yourself.

- ◯ Exclusively heterosexual
- ◯ Mostly heterosexual
- ◯ Bisexual -- that is, attracted to men and women equally
- ◯ Mostly gay/lesbian
- ◯ Exclusively gay/lesbian
- ◯ Not sexually attracted to either males or females
- ◯ I don't know
- ◯ I prefer not to answer

Have you been sexually active in the last four weeks?

- ◯ No
- ◯ Yes
- ◯ I prefer not to answer

Is your current primary partner the same person you were having sex with when you took the last survey?

- ◯ No
- ◯ Yes
- ◯ I prefer not to answer

What is the sex of your primary partner in the past 4 weeks?

- ◯ Male
- ◯ Female
- ◯ Something else
- ◯ I don't know
- ◯ I prefer not to answer

On a scale of 1 to 100, how might you rank your sex life right now?

worst possible — best possible

(Place a mark on the scale above)

What do you think would have to change to bring it to a 100?

____

In the last 4 weeks, would you say that your contraceptive method has...

- ◯ Improved my sex life a lot
- ◯ Improved my sex life a little
- ◯ Has had no effect on my sex life
- ◯ Has made my sex life a little worse
- ◯ Has made my sex life a lot worse

Please explain the impact your current contraceptive method has on your sex life.

____

Do you have any concern about your sexual functioning?

- ◯ No
- ◯ Yes
- ◯ I don't know

Please briefly describe.

____

We will now ask you a few questions about your sexual feelings and responses during the past four weeks. Just do your best to answer each one.

Over the past 4 weeks, how would you rate your level (degree) of sexual desire or interest?	◯ Very high<br>◯ High<br>◯ Moderate<br>◯ Low<br>◯ Very low or none at all<br>◯ Prefer not to answer
Over the past 4 weeks, how would you rate your level of sexual arousal ("turn on") during sexual activity or intercourse?	◯ No sexual activity<br>◯ Very high<br>◯ High<br>◯ Moderate<br>◯ Low<br>◯ Very low or none at all<br>◯ Prefer not to answer
Over the past 4 weeks, how often did you become lubricated ("wet") during sexual activity or intercourse?	◯ No sexual activity<br>◯ Almost always or always<br>◯ Most times (more than half the time)<br>◯ Sometimes (about half the time)<br>◯ A few times (less than half the time)<br>◯ Almost never or never<br>◯ Prefer not to answer
Over the past 4 weeks, when you had sexual stimulation or intercourse, how often did you reach orgasm (climax)?	◯ No sexual activity<br>◯ Almost always or always<br>◯ Most times (more than half the time)<br>◯ Sometimes (about half the time)<br>◯ A few times (less than half the time)<br>◯ Almost never or never<br>◯ Prefer not to answer
Over the past 4 weeks, how satisfied have you been with your overall sexual life?	◯ Very satisfied<br>◯ Moderately satisfied<br>◯ About equally satisfied and dissatisfied<br>◯ Moderately dissatisfied<br>◯ Very dissatisfied<br>◯ Prefer not to answer
Over the past 4 weeks, how often did you experience discomfort or pain during vaginal penetration?	◯ Did not attempt intercourse<br>◯ Almost always or always<br>◯ Most times (more than half the time)<br>◯ Sometimes (about half the time)<br>◯ A few times (less than half the time)<br>◯ Almost never or never<br>◯ Prefer not to answer

The following items relate to your sexual experiences. You might find a few of the items similar to items you just filled out. That's okay; just do your best to answer all the questions.

When responding to these items, please think about the last 4 weeks and how satisfied or dissatisfied are you with each of the following issues.

	not at all satisfied	a little satisfied	moderately satisfied	very satisfied	extremely satisfied	I prefer not to answer
The intensity of my sexual arousal	○	○	○	○	○	○
The quality of my orgasms	○	○	○	○	○	○
My letting go and surrender to sexual pleasure during sex	○	○	○	○	○	○
My focus and concentration during sexual activity	○	○	○	○	○	○
The way I sexually react to my partner	○	○	○	○	○	○
My body's sexual functioning	○	○	○	○	○	○
My emotional opening up in sex	○	○	○	○	○	○
My mood after sexual activity	○	○	○	○	○	○
The frequency of my orgasms	○	○	○	○	○	○
The pleasure I provide to my partner	○	○	○	○	○	○
The balance between what I give and receive in sex	○	○	○	○	○	○
My partner's emotional opening up during sex	○	○	○	○	○	○
My partner's initiation of sexual activity	○	○	○	○	○	○
My partner's ability to orgasm	○	○	○	○	○	○
My partner's surrender to sexual pleasure (letting go)	○	○	○	○	○	○
The way my partner takes care of my sexual needs	○	○	○	○	○	○
My partner's sexual creativity	○	○	○	○	○	○
My partner's sexual availability	○	○	○	○	○	○
The variety of my sexual activities	○	○	○	○	○	○
The frequency of my sexual activity	○	○	○	○	○	○

The last few items have to do with more general health and well-being. Please think about your experiences in the last four weeks when answering.

	All of the time	Most of the time	More than half of the time	Less than half of the time	Some of the time	At no time
I have felt cheerful and in good spirits	○	○	○	○	○	○
I have felt calm and relaxed	○	○	○	○	○	○
I have felt active and vigorous	○	○	○	○	○	○
I woke up feeling fresh and rested	○	○	○	○	○	○
My daily life has been filled with things that interest me	○	○	○	○	○	○

Thank you again for participating in this study. In the space below, feel free to tell us anything else you wish about your experience with your contraception or participation in this study. Otherwise, we will be in touch with you when it's time for the next part of the study.

Comments: ____

Brief Followup Survey (1, 2, & 3 years)

Welcome Back! As you'll remember, this study is helping us learn more about relationships between people's birth control experiences. As researchers, we have much more to learn about this topic, and so what we learn in this survey will be very helpful to us. Findings could help improve the quality of reproductive health care.

We estimate that this survey will take you about 10 minutes to complete. Once you complete the survey, you will receive a $20 gift card credit as a thank you for your time.

Do your best to answer each question, and remember that all information is completely confidential.

Thank you warmly for your participation.

Thank you very much for participating in this study.

First we want to make sure you haven't changed any of your contact information.

In the past six months, has any of your contact information changed (that is, your phone number, email, or mailing address)?	◯ No<br>◯ Yes
If so, which contact info has changed?	☐ New phone number<br>☐ New email address<br>☐ New home address<br>(check all that apply)
Please enter in your new phone number.	__________________________<br>(xxx-xxx-xxxx)
Please enter in your new email address.	__________________________
Please enter in your new mailing address.	________________________________

The following questions are about your pregnancy plans and recent changes in contraception.

What method(s) to prevent pregnancy have you used in the last 4 weeks?

- ☐ Contraceptive Implant (Nexplanon)
- ☐ Copper IUD (Paragard)
- ☐ Hormonal IUD (Mirena)
- ☐ Hormonal IUD (Liletta)
- ☐ Other hormonal IUD (Skyla)
- ☐ Injection (Depo-Provera)
- ☐ Combined oral contraceptive pill (The Pill)
- ☐ Progestin Only Pill (Minipill)
- ☐ Contraceptive patch (Xulane or OrthoEvra)
- ☐ Vaginal ring (NuvaRing)
- ☐ Male condom
- ☐ Female condom
- ☐ Cervical cap or sponge
- ☐ Spermicide
- ☐ Diaphragm
- ☐ Fertility Awareness Method / Natural Family Planning / Rhythm Method
- ☐ Withdrawal
- ☐ Levonorgestrel Emergency Contraception (Plan B/Next Choice)
- ☐ Ulipristal EC Pill (Ella)
- ☐ Other
- ☐ None; I am not using any contraceptive method

(select all that apply)

If other, please specify:

____

Are you still using the birth control method that you received at the beginning of this study?

- ◯ No
- ◯ Yes

In the last 4 weeks, have you checked to make sure your IUD or implant is still in place?

- ◯ No, I have not
- ◯ Yes, I felt my strings (or rod in my arm) myself and confirmed it's still there
- ◯ Yes, I had a provider check
- ◯ I tried but was not able to feel anything

How long do you plan on using the birth control method you are using?

- ◯ Less than 1 year
- ◯ More than 1 year but less than 2 years
- ◯ More than 2 years but less than 3 years
- ◯ More than 3 years but less than 5 years
- ◯ More than 5 years but less than 10 years
- ◯ More than 10 years
- ◯ Unsure
- ◯ I prefer not to answer this question

What are the reasons you are no longer using the method you started at the beginning of the study?	☐ Bleeding issues (spotting, irregular, ect)<br>☐ Excessive bleeding<br>☐ Cramping<br>☐ Pain<br>☐ Breast symptoms<br>☐ Weight gain<br>☐ Weight loss<br>☐ Moodiness or depression<br>☐ Bloating<br>☐ Skin problems<br>☐ Loss of libido/sexual interest<br>☐ Pain during intercourse<br>☐ Partner complaint<br>☐ IUD fell out/expelled<br>☐ I wanted to get pregnant<br>☐ I had a positive pregnancy test<br>☐ Other
If other, please specify:	______________________________
What was the date the device fell out or was removed?<br><br>If you can't remember the exact date, just make your best guess.	______________________________
When did you start using your new method?	______________________________<br>(please make you best guess)
Overall, how satisfied are you with the method(s) you were using during the last 4 weeks?	◯ Completely satisfied<br>◯ Somewhat satisfied<br>◯ Neither satisfied or dissatisfied<br>◯ Somewhat dissatisfied<br>◯ Completely dissatisfied<br>◯ I prefer not to answer
Overall, how confident were you that the method(s) you have been using the last 4 weeks will prevent pregnancy?	◯ Very high confidence<br>◯ High confidence<br>◯ Moderate confidence<br>◯ Low confidence<br>◯ Very low or no confidence<br>◯ I prefer not to answer
Please rate your agreement or disagreement with the following statement: "I feel that I have control over whether or not I get pregnant."	◯ I strongly agree<br>◯ I somewhat agree<br>◯ I neither agree nor disagree<br>◯ I somewhat disagree<br>◯ I strongly disagree<br>◯ I prefer not to answer
In the last 4 weeks, would you say that your birth control or method to avoid pregnancy has:	◯ Improved my sex life a lot<br>◯ Improved my sex life a little<br>◯ Has had no effect on my sex life<br>◯ Has made my sex life a little worse<br>◯ Has made my sex life a lot worse

Briefly explain the impact your method used in the last 4 weeks to avoid pregnancy has on your sex life.

____

Now we would like to ask you about your plans for future children, ideal timing, and feelings surrounding pregnancy.

Do you think you would like to have children (or more children) in the future?

- ◯ No
- ◯ Yes
- ◯ I don't know

What are your future pregnancy plans?

- ◯ I do not plan on getting pregnant at any time in the future.
- ◯ I am currently trying to get pregnant.
- ◯ I would like to get pregnant in the next year.
- ◯ I would like to get pregnant in the next 2-5 years (but not this year).
- ◯ I would like to get pregnant in the next 5-10 years but not before then.
- ◯ Unsure
- ◯ Other

What are your pregnancy plans?

____

How important is it to you to not get pregnant until you are ready?

not at all important — the most important

(Place a mark on the scale above)

How important is it to you to not get pregnant now or in the future?

not at all important — the most important

(Place a mark on the scale above)

How would you feel if you got pregnant in the next month?

worst feeling you can imagine — happiest you could possibly feel

(Place a mark on the scale above)

Please tell us a bit more about how a pregnancy now or in the near future would affect your life.

____

Which of the following best describes your religious identity?

- ◯ Not Religious
- ◯ Christian (Protestant, Evangelical, Mainline, etc)
- ◯ Catholic
- ◯ Mormon
- ◯ Jewish
- ◯ Muslim
- ◯ Other
- ◯ Don't know or prefer not to answer

Please describe your religious identity.

____

Please tell us a bit more about yourself. None of your answers will impact any services you may receive.

We understand that not everyone who needs or wants birth control identifies as a woman.

Please select the word(s) you use to describe yourself, or select self-describe and you can write in the word(s) you use.

- ☐ Woman
- ☐ Man
- ☐ Non-binary
- ☐ Transgender
- ☐ Prefer to self-describe

(check all that apply)

Please describe yourself.

____

Please choose the description that best fits how you think of yourself.

- ◯ Exclusively Heterosexual (Straight)
- ◯ Mostly Heterosexual
- ◯ Bisexual (Attracted to both Men and Women)
- ◯ Mostly Gay/Lesbian
- ◯ Exclusively Homosexual (Gay/Lesbian)
- ◯ Not sexually attracted to either males or females
- ◯ Other
- ◯ I don't know
- ◯ I prefer not to answer

In the last 12 months have you had sex with...

- ◯ Not currently sexually active
- ◯ Males only
- ◯ Both males and females
- ◯ Females only
- ◯ I prefer not to answer

Which of the following best describes your current relationship situation?

- ◯ Married
- ◯ Not married, but living together or in a committed relationship
- ◯ Actively dating, but NOT in a committed relationship
- ◯ Divorced/Separated
- ◯ Single, not in a relationship
- ◯ Widowed
- ◯ Other
- ◯ I prefer not to answer

What type of medical insurance do you currently have?

- ◯ None
- ◯ Medicaid
- ◯ Insurance through your job or that you buy on your own
- ◯ Student health insurance
- ◯ Parent's insurance
- ◯ Military or VA (Champus, ChampVA, Tricare)
- ◯ Disability or Medicare
- ◯ I don't know
- ◯ I prefer not to answer

What best describes your current employment status?

- ☐ Unemployed
- ☐ Working full-time (at least 30 hours/week)
- ☐ Working part-time (less than 30 hours/week)
- ☐ Disabled, sick leave, family leave
- ☐ Retired
- ☐ Homemaker
- ☐ Student
- ☐ Other
- ☐ I prefer not to answer

If other employment, please describe.

____

How many hours do you work each week?

____
(Please guess if you are not sure)

What is your hourly wage?

____

What kind of work do you do?

____

Are you currently looking for work, additional work, or different work?

◯ No
◯ Yes
◯ Don't Know
◯ I prefer not to answer this question

Were you incarcerated in jail or prison in the last 12 months? (Even if only for a few hours or days)

◯ No
◯ Yes
◯ I prefer not to answer

How many times were you incarcerated in the last 12 months? (Even if only for a few hours or days)

◯ 1
◯ 2
◯ 3
◯ 4
◯ 5
◯ 6
◯ 7
◯ 8
◯ 9
◯ 10
◯ 11
◯ 12 or more

Please list the dates you were incarcerated (your best guess is fine) and where.

example
1) 1/15/16-1/20/16, Salt Lake County Jail
2) 2/5/16-4/5/16, Utah State Prision

____

Were you able to continue using your birth control while you were incarcerated?

◯ Yes
◯ No

Please describe the ways your incarceration impacted your use of birth control or pregnancy plans.

____

The next few questions are about your education.

What best describes the highest level of education you have COMPLETED SO FAR?

- ◯ 11th grade or less
- ◯ 12th grade (completed high school or GED)
- ◯ Vocational/technical training
- ◯ Associate degree or some college
- ◯ 4-year college degree (BA/BS)
- ◯ Any graduate or professional education (any time in a Masters, JD, PhD, MD, etc. program)
- ◯ I don't know
- ◯ I prefer not to answer

Are you currently in school, either full-time or part-time?

- ◯ Not at all
- ◯ Part-time
- ◯ Full-time
- ◯ I prefer not to answer this question

What type of degree are you seeking?

- ◯ I plan to finish high school or GED
- ◯ Get vocational/technical training
- ◯ Get an associate degree
- ◯ Get a 4-year college degree (BA/BS)
- ◯ Get graduate or professional education (Masters, JD PhD, MD)
- ◯ I don't know
- ◯ I prefer not to answer

Please specify type

____

What date do you expect to graduate?

____

What best describes your PLANS for the highest level of education you hope to achieve in the future?

- ◯ None, I am done with school and do not have plans to go back
- ◯ I plan to finish high school or GED
- ◯ Get vocational/technical training
- ◯ Get an associate degree
- ◯ Get a 4-year college degree (BA/BS)
- ◯ Get graduate or professional education (Masters, JD PhD, MD)
- ◯ I don't know
- ◯ I prefer not to answer

Just a few more questions about your financial situation.

What is your annual household income?

- ◯ Less than $10,000
- ◯ $10,000-$19,999
- ◯ $20,000-$29,999
- ◯ $30,000-$39,999
- ◯ $40,000-$49,999
- ◯ $50,000-$59,999
- ◯ $60,000-$69,999
- ◯ $70,000-$79,999
- ◯ $80,000 or more
- ◯ I don't know
- ◯ I prefer not to answer this question

What is your best estimate of your annual household income?

____

How many people are in your household?

- ◯ 1
- ◯ 2
- ◯ 3
- ◯ 4
- ◯ 5
- ◯ 6
- ◯ 7
- ◯ 8
- ◯ 9
- ◯ 10
- ◯ 11
- ◯ 12
- ◯ 13
- ◯ 14
- ◯ 15+

How many children under the age of 18 do you have living with you?

____

Please check all of the following that have been a source of income in the last month:

- ☐ Self
- ☐ Spouse or Partner
- ☐ Other family member
- ☐ Government assistance
- ☐ Other
- ☐ I prefer not to answer

(Check all that apply)

What other sources of income did you receive in the last month?

____

How much money did you make last month?

- ◯ None
- ◯ $1-$400
- ◯ $401-$800
- ◯ $801-$1,200
- ◯ $1,201-$1,600
- ◯ $1,601-$2,000
- ◯ $2,001-$2,400
- ◯ $2,401-$2,800
- ◯ More than $2,800
- ◯ Don't know
- ◯ I prefer not to answer

How much money did your partner or spouse make last month?	◯ None<br>◯ $1-$400<br>◯ $401-$800<br>◯ $801-$1,200<br>◯ $1,201-$1,600<br>◯ $1,601-$2,000<br>◯ $2,001-$2,400<br>◯ $2,401-$2,800<br>◯ More than $2,800<br>◯ Don't know<br>◯ I prefer not to answer
How much money did your other family members contribute to your household income last month?	◯ None<br>◯ $1-$400<br>◯ $401-$800<br>◯ $801-$1,200<br>◯ $1,201-$1,600<br>◯ $1,601-$2,000<br>◯ $2,001-$2,400<br>◯ $2,401-$2,800<br>◯ More than $2,800<br>◯ Don't know<br>◯ I prefer not to answer
How much money did government assistance contribute to your household income last month?	◯ None<br>◯ $1-$400<br>◯ $401-$800<br>◯ $801-$1,200<br>◯ $1,201-$1,600<br>◯ $1,601-$2,000<br>◯ $2,001-$2,400<br>◯ $2,401-$2,800<br>◯ More than $2,800<br>◯ Don't know<br>◯ I prefer not to answer
How much money did you receive from other sources last month?	◯ None<br>◯ $1-$400<br>◯ $401-$800<br>◯ $801-$1,200<br>◯ $1,201-$1,600<br>◯ $1,601-$2,000<br>◯ $2,001-$2,400<br>◯ $2,401-$2,800<br>◯ More than $2,800<br>◯ Don't know<br>◯ I prefer not to answer
Were you supposed to receive any child support in the last 4 weeks?	◯ No<br>◯ Yes<br>◯ I prefer not to answer
How much child support were you supposed to receive?	__________________________________
Did you receive any child support last 4 weeks?	◯ No<br>◯ Yes<br>◯ I prefer not to answer this question
How much child support did you receive in the last 4 weeks?	__________________________________

Which of the following best describes your current housing situation?	◯ Homeless<br>◯ Shelter<br>◯ Mobile home<br>◯ Apartment<br>◯ Single-family house<br>◯ Other<br>◯ I prefer not to answer this question
Describe type of housing:	______________________________

We will now ask a few questions about public assistance you may receive:

Do you currently receive:

	No	Yes	Prefer not to answer
Food stamps	○	○	○
WIC (Women, Infants and Children)	○	○	○
Welfare	○	○	○
Unemployment benefits	○	○	○

Just a few more questions about your economic situation.

During the past 12 months, have you had trouble paying for the following:

	No	Yes	Prefer not to answer
Transportation	◯	◯	◯
Housing	◯	◯	◯
Medical care or medications	◯	◯	◯
Food	◯	◯	◯

During the past month, how often would you say you had enough money to meet your basic living needs such as food, housing and transportation?

◯ All the time
◯ Most of the time
◯ Some of the time
◯ Rarely
◯ Never
◯ Don't Know
◯ Refused

The last few items have to do with your general health and well-being. Please think about the last 4 weeks when responding to these items.

	All of the time	Most of the time	More than half of the time	Less than half of the time	Some of the time	At no time
I have felt cheerful and in good spirits	○	○	○	○	○	○
I have felt calm and relaxed	○	○	○	○	○	○
I have felt active and vigorous	○	○	○	○	○	○
I woke up feeling fresh and rested	○	○	○	○	○	○
My daily life has been filled with things that interest me	○	○	○	○	○	○

The final questions are about any health issues that may be related to your contraception.

Have you had a positive pregnancy test since the last survey?
- ◯ No
- ◯ Yes
- ◯ I don't know

What was the date of your positive pregnancy test?

____

When you got pregnant, were you trying to get pregnant?
- ◯ Yes
- ◯ No

If you have had a positive pregnancy test please describe the outcome of the pregnancy.
- ◯ I had or am planning to have an abortion
- ◯ I had a miscarriage
- ◯ I had an ectopic pregnancy
- ◯ I am planning on continuing the pregnancy and keeping the baby
- ◯ I am planning on continuing the pregnancy and placing the baby up for adoption
- ◯ I am unsure of what I am going to do
- ◯ I have not had a positive pregnancy test

What was the date when your pregnancy ended, regardless of the outcome?

____

To ensure your safety, if you had a pregnancy, we would like to follow-up on the care you received. Please provide the name of the clinic or hospital where you were seen.

____

Since enrolling in the study, have you been hospitalized for any illness or injury?
- ◯ No
- ◯ Yes

Please provide the date:

____

Please explain what happened:

____

Have you seen a medical provider for an issue that you thought might be related to the IUD or contraceptive implant you had inserted?
- ◯ No
- ◯ Yes

Please provide the date:

____

Please describe:

____

Pharmacy Access

In 2018, Utah passed legislation that allows for pharmacists to dispense birth control pills, patches, and rings to women without a prescription from a doctor.

Were you aware of this law before now?

◯ Yes ◯ No

How comfortable do you feel talking about contraception with the following individuals?

	Very comfortable	Comfortable	Neutral	Uncomfortable	Very uncomfortable
Partner(s)	◯	◯	◯	◯	◯
Parent(s) or guardian(s)	◯	◯	◯	◯	◯
Friend(s)	◯	◯	◯	◯	◯
Pharmacist(s)	◯	◯	◯	◯	◯
Doctor(s)	◯	◯	◯	◯	◯
Teacher(s)	◯	◯	◯	◯	◯

Please rate you level of agreement with the following questions.

	Strongly agree	Agree	Neutral	Disagree	Strongly disagree
I am comfortable getting my birth control from a pharmacist without a doctor's prescription.	○	○	○	○	○
I believe that pharmacist are knowledgeable enough to counsel me on birth control.	○	○	○	○	○
I worry that my birth control counseling would be too public with a pharmacist.	○	○	○	○	○
I am more comfortable seeing a physician for birth control.	○	○	○	○	○
I want my pharmacist to counsel me on all of my birth control options.	○	○	○	○	○
I plan to get my birth control directly from a pharmacist without seeing a provider first in the next year.	○	○	○	○	○
Having the option to get birth control from a pharmacist without having to see a provider first will make birth control more accessible to me or someone I know.	○	○	○	○	○

What are the main reasons that you would not get birth control from a pharmacist?

____

What are the main reasons that you would get birth control from a pharmacist?

____

How much would you be willing to pay out-of-pocket for birth control from a pharmacy without having to see a provider first (per month)?

____
(Please enter in X.XX format)

How much would you be willing to pay out-of-pocket for a one-time pharmacy consulting fee?

____
(Please enter in X.XX format)

Thank you again for participating in this study.

Feel free to tell us anything about your experience with your contraception or participation in this study.

Comments: ______________________

Brief Followup Survey (18 &30 months)

Welcome Back! As you'll remember, this study is helping us learn more about relationship between people's birth control experiences. As researchers, we have much more to learn about this topic, and so what we learn in this survey will be very helpful to us. Findings could help improve the quality of reproductive health care.

We estimate that this survey will take you about 5 minutes to complete.

Do your best to answer each question, and remember that all information is completely confidential.

Thank you warmly for your participation.

Thank you very much for participating in this study. First we want to make sure you haven't changed any of your contact information.

Has any of your contact information changed in the last six months (for example, your telephone number, email, or mailing address)?	◯ No<br>◯ Yes
If so, which contact info has changed?	☐ New phone number<br>☐ New email address<br>☐ New home address<br>(check all that apply)
Please provide your new phone number.	________________________________<br>(xxx-xxx-xxxx)
Please provide your new email address.	________________________________
Please provide your new mailing address.	____

We will start by asking you a few questions about your birth control.

What method(s) to prevent pregnancy have you used in the last 4 weeks?	☐ Contraceptive Implant (Nexplanon)<br>☐ Copper IUD (Paragard)<br>☐ Hormonal IUD (Mirena)<br>☐ Hormonal IUD (Liletta)<br>☐ Other hormonal IUD (Skyla)<br>☐ Injection (Depo-Provera)<br>☐ Combined oral contraceptive pill (The Pill)<br>☐ Progestin Only Pill (Minipill)<br>☐ Contraceptive patch (Xulane or OrthoEvra)<br>☐ Vaginal ring (NuvaRing)<br>☐ Male condom<br>☐ Female condom<br>☐ Cervical cap or sponge<br>☐ Spermicide<br>☐ Diaphragm<br>☐ Fertility Awareness Method / Natural Family Planning / Rhythm Method<br>☐ Withdrawal<br>☐ Levonorgestrel Emergency Contraception (Plan B/Next Choice)<br>☐ Ulipristal EC Pill (Ella)<br>☐ Other<br>☐ None; I am not using any contraceptive method<br>(select all that apply)
If other, please specify:	__________________________________
Are you still using the same method of contraception that you received at the beginning of this study?	◯ Yes<br>◯ No
In the last 4 weeks, have you checked to make sure your IUD or implant is still in place?	◯ No, I have not<br>◯ Yes, I felt my strings (or rod in my arm) myself and confirmed it's still there<br>◯ Yes, I had a provider check<br>◯ I tried but was not able to feel anything
On what day did the device fall out or was removed?<br><br>If you can't remember the exact date, just make your best guess.	__________________________________
When did you start using your new method?	__________________________________

What are the reasons you are no longer using the method you received at the start of the study?

☐ Bleeding issues (spotting, irregular, ect)
☐ Excessive bleeding
☐ Cramping
☐ Pain
☐ Breast symptoms
☐ Weight gain
☐ Weight loss
☐ Moodiness or depression
☐ Bloating
☐ Skin problems
☐ Loss of libido/sexual interest
☐ Pain during intercourse
☐ Partner complaint
☐ IUD fell out/expelled
☐ I wanted to get pregnant
☐ I had a positive pregnancy test
☐ Other
(Check all that apply)

If other, please specify:

____

Now we want to ask you some questions about birth control. Please think about about all the method(s) you may have used recently, including the IUD or implant.

Overall, how satisfied are you with the method(s) you were using during the last 4 weeks?

◯ Completely satisfied
◯ Somewhat satisfied
◯ Neither satisfied or dissatisfied
◯ Somewhat dissatisfied
◯ Completely dissatisfied
◯ I prefer not to answer

Overall, how confident were you that the method you have been using the last 4 weeks will prevent pregnancy?

◯ Very high confidence
◯ High confidence
◯ Moderate confidence
◯ Low confidence
◯ Very low or no confidence
◯ I prefer not to answer

Please rate your agreement or disagreement with the following statement: "I feel that I have control over whether or not I get pregnant."

◯ I strongly agree
◯ I somewhat agree
◯ I neither agree nor disagree
◯ I somewhat disagree
◯ I strongly disagree
◯ I prefer not to answer

The final questions are about any pregnancies or health issues that may be related to your contraception.

Have you had a positive pregnancy test since the last survey? (That is, in the last six months?)

◯ No
◯ Yes
◯ I don't know

What was the date of your positive pregnancy test?

____

When you got pregnant, were you trying to get pregnant?

◯ Yes
◯ No

If you have had a positive pregnancy test please describe the outcome of the pregnancy.	◯ I had or am planning to have an abortion<br>◯ I had a miscarriage<br>◯ I had an ectopic pregnancy<br>◯ I am planning on continuing the pregnancy and keeping the baby<br>◯ I am planning on continuing the pregnancy and giving the baby up for adoption<br>◯ I am unsure of what I am going to do
To ensure your safety, if you had a pregnancy, we would like to follow-up on the care you received. Please provide the name of the clinic or hospital where you were seen.	__________________________________
Since enrolling in the study, have you been hospitalized for any illness or injury?	◯ No<br>◯ Yes
Please provide the date:	___________________________
Please describe what happened:	__________________________________
Have you seen a medical provider for an issue that you thought might be related to the IUD or contraceptive implant you had inserted?	◯ No<br>◯ Yes
Please provide the date:	___________________________
Please describe:	__________________________________

Pharmacy Access

In 2018, Utah passed legislation that allows for pharmacists to dispense birth control pills, patches, and rings to women without a prescription from a doctor.

Were you aware of this law before now?

◯ Yes ◯ No

How comfortable do you feel talking about contraception with the following individuals?

	Very comfortable	Comfortable	Neutral	Uncomfortable	Very uncomfortable
Partner(s)	○	○	○	○	○
Parent(s) or guardian(s)	○	○	○	○	○
Friend(s)	○	○	○	○	○
Pharmacist(s)	○	○	○	○	○
Doctor(s)	○	○	○	○	○
Teacher(s)	○	○	○	○	○

Please rate you level of agreement with the following questions.

	Strongly agree	Agree	Neutral	Disagree	Strongly disagree
I am comfortable getting my birth control from a pharmacist without a doctor's prescription.	○	○	○	○	○
I believe that pharmacist are knowledgeable enough to counsel me on birth control.	○	○	○	○	○
I worry that my birth control counseling would be too public with a pharmacist.	○	○	○	○	○
I am more comfortable seeing a physician for birth control.	○	○	○	○	○
I want my pharmacist to counsel me on all of my birth control options.	○	○	○	○	○
I plan to get my birth control directly from a pharmacist without seeing a provider first in the next year.	○	○	○	○	○
Having the option to get birth control from a pharmacist without having to see a provider first will make birth control more accessible to me or someone I know.	○	○	○	○	○

What are the main reasons that you would not get birth control from a pharmacist?

____

What are the main reasons that you would get birth control from a pharmacist?

____

How much would you be willing to pay out-of-pocket for birth control from a pharmacy without having to see a provider first (per month)?

____
(Please enter in X.XX format)

How much would you be willing to pay out-of-pocket for a one-time pharmacy consulting fee?

____
(Please enter in X.XX format)

Thank you again for participating in this study. In the space below, feel free to tell us anything about your experience with your contraception or participation in this study. Otherwise, we will be in touch in the future.

Comments:

____

Primer Cuestionario

¡Bienvenida! A pesar de que 99% de mujeres usan un método anticonceptivo en algún momento de su vida, todavía tenemos mucho que aprender. Este estudio nos ayudará a conocer más en donde las personas encuentran información sobre métodos anticonceptivos, y también más sobre la relación que hay entre el método anticonceptivo y las prácticas sexuales de las personas. Estos resultados podrían ayudar a mejorar la calidad de la salud reproductiva.

Calculamos que esta encuesta le tomará 15-30 minutos para completarla. Cuando la termine, usted recibirá una tarjeta de regalo de $20 para agradecerle por su tiempo.

Haga lo mejor que pueda al contestar cada pregunta, y por favor recuerde que toda esta información es completamente confidencial.

Le agradecemos calurosamente por su participación.

Nos gustaría hacerle unas preguntas sobre su visita en la clínica hoy.

¿Cuál es la razón principal por la que vino a la clínica hoy?

- ☐ Examen anual, incluyendo Papanicolaou
- ☐ Para obtener un método anticonceptivo
- ☐ Para obtener método anticonceptivo de EMERGENCIA
- ☐ Para una prueba de embarazo
- ☐ Para un examen de enfermedades sexuales
- ☐ Para servicios de aborto
- ☐ Para obtener un método anticonceptivo gratis o de bajo costo
- ☐ Para participar en este estudio
- ☐ Prefiero no contestar esta pregunta
- ☐ Otra razón

Si es otra razón, por favor de describir la razón por haber venido a la clínica hoy:

____

¿Cómo se enteró de los servicios que esta clínica proporciona?

- ☐ Familiar, amigo(a), o mi pareja
- ☐ Clase en la escuela o maestro(a)
- ☐ Proveedor médico u otra clínica
- ☐ Facebook/Twitter u otras redes sociales
- ☐ Bedsider.org
- ☐ Otra página en la internet
- ☐ Televisión, radio, o periódico
- ☐ Plannedparenthood.org
- ☐ Otro
- ☐ Yo he venido a la clínica antes
- ☐ No lo sé
- ☐ Prefiero no contestar esta pregunta

(Marque todas las respuestas que correspondan)

¿Cómo se enteró de los servicios que esta clínica proporciona?

- ☐ Familiar, amigo(a)
- ☐ Mi pareja, novio(a), esposo(a)
- ☐ Clase en la escuela o maestro(a)
- ☐ Proveedor médico u otra clínica
- ☐ Facebook/Twitter u otras redes sociales
- ☐ Publicidad de Planned Parenthood
- ☐ www.Bedsider.org
- ☐ Otra página en la internet
- ☐ Televisión, radio, o periódico
- ☐ Yo he venido a la clínica antes
- ☐ Mensaje enviado directamente a mi teléfono
- ☐ www.hersaltlake.org
- ☐ Otro
- ☐ No lo sé
- ☐ Prefiero no contestar esta pregunta

(Marque todas las respuestas que correspondan)

Si hay otra razon, por favor de describir cómo se enteró sobre los sevicios que ofrecemos:

____

¿Alguna vez ha visitado la página de internet www.hersaltlake.org?

- ◯ No
- ◯ Sí
- ◯ No lo sé
- ◯ Prefiero no contestar

¿Dónde se enteró de la página de la internet www.hersaltlake.org?	☐ Boca a boca (familia o amigas)<br>☐ Anuncio publicitario en la red (Internet)<br>☐ Facebook<br>☐ Instagram<br>☐ Twitter<br>☐ Periódico o radio<br>☐ Otro
¿Alguna vez ha visitado la página de internet www.proutah.org?	○ No<br>○ Sí<br>○ No lo sé<br>○ Prefiero no contestar
¿Dónde ha visto publicidad para Planned Parenthood o se ha enterado de proutah.org?	☐ Publicidad en la internet<br>☐ Medios de transportes públicos<br>☐ Carteles<br>☐ Radio<br>☐ Redes sociales<br>☐ Otro
¿Alguna vez usted ha visitado la página de internet www.bedsider.org?	○ No<br>○ Sí<br>○ No lo sé<br>○ Prefiero no contestar esta pregunta
Si usted ha visitado la página de internet www.bedsider.org, ¿Cómo es que usted supo de la página?	☐ Familiar, amigo(a)<br>☐ Mi pareja o esposo(a)<br>☐ Clase en la escuela o maestro(a)<br>☐ Proveedor médico u otra clínica<br>☐ Facebook/Twitter u otras redes sociales<br>☐ Cartelero o volante<br>☐ Otra página de internet<br>☐ Mensaje enviado directamente a mi teléfono<br>☐ Televisión, radio, o periódico<br>☐ Otro<br>☐ He estado en la clínica antes<br>☐ No lo sé<br>☐ Prefiero no contestar esta pregunta<br>(Marque todas las respuestas que correspondan)
Si usted ha visitado la página de internet www.bedsider.org, ¿Cómo es que usted supo de la página?	☐ Familiar, amigo(a)<br>☐ Mi pareja, novio(a), o esposo(a)<br>☐ Clase en la escuela o maestro(a)<br>☐ Proveedor médico u otra clínica<br>☐ Facebook/Twitter u otras redes sociales<br>☐ www.Bedsider.org<br>☐ Otra página de internet<br>☐ Mensaje enviado directamente a mi teléfono<br>☐ www.hersaltlake.org<br>☐ Televisión, radio, o periódico<br>☐ Otro<br>☐ He estado en la clínica antes<br>☐ No lo sé<br>☐ Prefiero no contestar esta pregunta<br>(Marque todas las respuestas que correspondan)
Si hay otro, por favor de describirlo:	__________________________

Antecedentes del participante

Primero, por favor díganos un poco sobre usted. Sus respuestas no impactarán su participación en el estudio, su cuidado clínico, o cualquier otro servicio que recibe.

¿Nació en los Estados Unidos?

- ◯ No
- ◯ Sí
- ◯ Prefiero no contestar esta pregunta

¿En qué país nació?

____

¿En qué año se movió a los Estados Unidos?

____

Reconocemos que no todas las personas que quieran o necesiten anticoncepción se identifican como mujeres.

Para la siguiente pregunta, por favor seleccione las palabras que usted utiliza para describirse personalmente, o escriba en sus propias palabras como se identifica.

- ☐ Mujer
- ☐ Hombre
- ☐ No binario
- ☐ Transgénero
- ☐ Prefiero auto-describirme

Escriba en sus propias palabras como se identifica?

____

¿Cuál de los siguientes mejor describe su etnia/raza?

- ☐ Blanca o Anglosajona
- ☐ Hispana o Latina
- ☐ Asiática
- ☐ Nativa de Hawái o de Otras Islas del Pacífico
- ☐ India Americana o Nativa de Alaska
- ☐ Negra o Afroamericana
- ☐ Otro
- ☐ No lo sé
- ☐ Prefiero no contestar esta pregunta

Si hay otro, por favor de describirlo:

____

¿Cuál de los siguientes mejor describe su identidad religiosa?

- ◯ No religiosa
- ◯ Cristiana (Protestante, Evangélica, etc.)
- ◯ Católica
- ◯ Mormona
- ◯ Judía
- ◯ Musulmán
- ◯ Otro
- ◯ No lo sé o prefiero no contestar

Por favor describe su identidad religiosa.

____

¿Cuál de los siguientes mejor describe su estado civil actual?	◯ Casada<br>◯ Soltera, pero vivo con mi pareja o en una relación de compromiso<br>◯ Citas , pero no en una relación de compromiso<br>◯ Separada o Divorciada<br>◯ Soltera (nunca he estado casada)<br>◯ Viuda<br>◯ Otro<br>◯ Prefiero no contestar esta pregunta
Si hay otro, por favor de describirlo:	__________________________________
¿Qué es lo que mejor describe su identidad sexual?	◯ Heterosexual (Straight)<br>◯ Homosexual (Gay/Lesbiana)<br>◯ Bisexual<br>◯ Otro<br>◯ No lo sé<br>◯ Prefiero no contestar esta pregunta
Por favor escoja la descripción sobre su identidad sexual que más ajusta a lo que usted siente de sí misma.	◯ Exclusivamente heterosexual<br>◯ Mayormente heterosexual<br>◯ Bisexual - atraída a hombres y mujeres igualmente<br>◯ Mayormente gay/lesbiana<br>◯ No atraída ni a hombres ni mujeres<br>◯ No sé<br>◯ Prefiero no contestar
En los últimos 12 meses, ha tenido sexo con:	◯ Actualmente no activa sexualmente<br>◯ Solo hombres<br>◯ Hombres y mujeres<br>◯ Solo mujeres<br>◯ Prefiero no contestar
Considerando todos los tipos de actividades sexuales, ¿con cuántas parejas femeninas alguna vez ha tenido sexo?	__________________________________
Considerando todos los tipos de actividades sexuales, ¿con cuántas parejas femeninas alguna vez ha tenido sexo en los últimos 12 meses aun si solo fue una vez?	__________________________________
¿Qué tipo de seguro médico tiene usted en este momento?	☐ Ninguno<br>☐ Medicaid<br>☐ Invalidez o Medicare<br>☐ Seguro médico del trabajo o que se compra por propia cuenta<br>☐ Militar o Veteranos (Champus, ChampVA, Tricare)<br>☐ Seguro médico de estudiante<br>☐ Seguro por parte de mis padres<br>☐ No lo sé<br>☐ Prefiero no contestar esta pregunta

¿Qué tipo de seguro médico tiene usted en este momento?	◯ Ninguno<br>◯ Medicaid<br>◯ Seguro médico del trabajo o que se compra por propia cuenta<br>◯ Seguro médico de estudiante<br>◯ Seguro por parte de mis padres<br>◯ Militar o Veteranos (Champus, ChampVA, Tricare)<br>◯ Invalidez o Medicare<br>◯ No lo sé<br>◯ Prefiero no contestar esta pregunta

Las siguientes preguntas son sobre su situación de empleo actual

¿Qué es lo que mejor describe su situación de empleo actual?

- ☐ Desempleada
- ☐ Empleada de tiempo completo (mínimo 30 horas/semana)
- ☐ Empleada de medio tiempo (menos de 30 horas/semana
- ☐ Invalidez o en permiso por enfermedad
- ☐ Jubilada
- ☐ Ama de casa
- ☐ Estudiante
- ☐ Otro
- ☐ Prefiero no contestar esta pregunta

(Marque todas las respuestas que correspondan)

Si su situación de empleo es diferente, por favor de describirlo:

____

¿Cuántas horas trabaja a la semana?

____

(Su mejor cálculo está bien)

¿Cuánto gana por hora?

____

(Ex. 7.25 si gana $7.25 por hora...Su mejor cálculo está bien)

¿Qué tipo de trabajo hace?

____

¿En este momento está buscando trabajo, ya sea trabajo adicional o diferente trabajo?

- ◯ No
- ◯ Sí
- ◯ No lo sé
- ◯ Prefiero no contestar esta pregunta

Las siguientes preguntas son sobre su nivel de educación y el nivel de educación de sus padres.

¿Qué es lo que mejor describe el nivel más alto de educación que usted ha TERMINADO hasta ahora?

- ◯ Grado 11 o menos
- ◯ Grado 12 (terminé el bachillerato o GED)
- ◯ Educación vocacional/técnica
- ◯ Título de 2 años o algo de Universidad
- ◯ Título de universidad (4 años)
- ◯ Título posgrado (maestría, doctorado, JD, MD, etc.)
- ◯ No lo sé
- ◯ Prefiero no contestar esta pregunta

¿Está usted actualmente en la escuela, ya sea a tiempo completo o medio tiempo?

- ◯ No en este momento
- ◯ Medio tiempo
- ◯ Tiempo completo
- ◯ Prefiero no contestar esta pregunta

¿Qué es lo que mejor describe sus PLANES para obtener el nivel más alto de educación en el futuro?

- ◯ Ninguno, ya terminé la escuela y no tengo planes de regresar
- ◯ Tengo planes de terminar el bachillerato o GED
- ◯ Pienso obtener una educación vocacional/técnica
- ◯ Obtener un título de 2 años
- ◯ Obtener un título de universidad (4 años)
- ◯ Obtener una educación de posgrado (maestría, doctorado, JD, MD, etc.)
- ◯ No lo sé
- ◯ Prefiero no contestar esta pregunta

¿En qué fecha espera graduarse?

____

¿Cuál es el nivel más alto de educación que su madre (o su guardiana) ha terminado?

- ◯ Grado 11 o menos
- ◯ Grado 12 (terminó el bachillerato o GED
- ◯ Educación vocacional/técnica
- ◯ Título de 2 años o algo de Universidad
- ◯ Título de universidad (4 años)
- ◯ Título posgrado (maestría, doctorado, JD, MD, etc.)
- ◯ No lo sé
- ◯ Prefiero no contestar esta pregunta

¿Cuál es el nivel más alto de educación que su padre (o su guardián) ha terminado

- ◯ Grado 11 o menos
- ◯ Grado 12 (terminó el bachillerato o GED
- ◯ Educación vocacional/técnica
- ◯ Título de 2 años o algo de Universidad
- ◯ Título de universidad (4 años)
- ◯ Título posgrado (maestría, doctorado, JD, MD, etc.)
- ◯ No lo sé
- ◯ Prefiero no contestar esta pregunta

Sólo unas pocas preguntas más sobre su situación financiera.

¿Cuál es el ingreso anual de su familia?

- ◯ Menos de $10,000
- ◯ $10,000-$19,999
- ◯ $20,000-$29,999
- ◯ $30,000-$39,999
- ◯ $40,000-$49,999
- ◯ $50,000-$59,999
- ◯ $60,000-$69,999
- ◯ $70,000-$79,999
- ◯ $80,000 o más
- ◯ No lo sé
- ◯ Prefiero no contestar esta pregunta

¿Cuál es su mejor cálculo de su ingreso anual del hogar ?

____
(8500 si ingreso anual es $8,500)

¿Cuántas personas viven en su hogar?

- ◯ 1
- ◯ 2
- ◯ 3
- ◯ 4
- ◯ 5
- ◯ 6
- ◯ 7
- ◯ 8
- ◯ 9
- ◯ 10
- ◯ 11
- ◯ 12
- ◯ 13
- ◯ 14
- ◯ 15+

(incluyendo as si misma)

¿Cuántos niños menores de 18 años viven con usted en su casa?

____

Por favor marque las siguientes opciones que han sido fuentes de ingreso en el último mes:

- ☐ Yo sola
- ☐ Mi esposo/pareja
- ☐ Mis familiares
- ☐ Asistencia de gobierno
- ☐ Otro
- ☐ Prefiero no contestar esta pregunta

(Marque todas las respuestas que correspondan)

¿Cuáles fueron otras fuentes de ingresos en el último mes?

____

¿Cuánto dinero ganó durante el mes pasado?	◯ Nada<br>◯ $1-$400<br>◯ $401-$800<br>◯ $801-$1,200<br>◯ $1,201-$1,600<br>◯ $1,601-$2,000<br>◯ $2,001-$2,400<br>◯ $2,401-$2,800<br>◯ Más de $2,800<br>◯ No lo sé<br>◯ Prefiero no contestar esta pregunta
¿Cuánto dinero ganó su pareja o conyuge durante el mes pasado?	◯ Nada<br>◯ $1-$400<br>◯ $401-$800<br>◯ $801-$1,200<br>◯ $1,201-$1,600<br>◯ $1,601-$2,000<br>◯ $2,001-$2,400<br>◯ $2,401-$2,800<br>◯ Más de $2,800<br>◯ No lo sé<br>◯ Prefiero no contestar esta pregunta
¿Cuánto dinero contribuyeron otros miembros de su familia/familiares para el ingreso familiar durante el mes pasado?	◯ Nada<br>◯ $1-$400<br>◯ $401-$800<br>◯ $801-$1,200<br>◯ $1,201-$1,600<br>◯ $1,601-$2,000<br>◯ $2,001-$2,400<br>◯ $2,401-$2,800<br>◯ Más de $2,800<br>◯ No lo sé<br>◯ Prefiero no contestar esta pregunta
¿Cuánto dinero recibió por parte de asistencia del gobierno para su ingreso familiar durante el mes pasado?	◯ Nada<br>◯ $1-$400<br>◯ $401-$800<br>◯ $801-$1,200<br>◯ $1,201-$1,600<br>◯ $1,601-$2,000<br>◯ $2,001-$2,400<br>◯ $2,401-$2,800<br>◯ Más de $2,800<br>◯ No lo sé<br>◯ Prefiero no contestar esta pregunta
¿Cuánto dinero recibió de otros recursos durante el mes pasado?	◯ Nada<br>◯ $1-$400<br>◯ $401-$800<br>◯ $801-$1,200<br>◯ $1,201-$1,600<br>◯ $1,601-$2,000<br>◯ $2,001-$2,400<br>◯ $2,401-$2,800<br>◯ Más de $2,800<br>◯ No lo sé<br>◯ Prefiero no contestar esta pregunta

¿Debió de haber recibido manutención de menores en las últimas 4 semanas?	◯ No<br>◯ Sí<br>◯ Prefiero no contestar esta pregunta
¿Cuánto dinero debió de haber recibido?	__________________________
¿Recibió dinero para manutención de menores durante las últimas 4 semanas?	◯ No<br>◯ Sí<br>◯ Prefiero no contestar esta pregunta
¿Cuánto dinero recibió para manutención de menores durante las últimas 4 semanas?	__________________________
¿Cuál de las siguientes opciones describe mejor su situación de vivienda actual?	◯ Sin vivienda (homeless)<br>◯ Refugio (shelter)<br>◯ Casa rodante (trailer)<br>◯ Apartamento<br>◯ Casa (solo una familia)<br>◯ Otro<br>◯ Prefiero no responder a esta pregunta
Describa que tipo de vivienda:	__________________________

Ahora vamos a hacerle algunas preguntas sobre la asistencia pública que usted ha estado recibiendo:

Actualmente usted recibe:

	No	Sí	Prefiero no contestar esta pregunta
Estampillas de comida	○	○	○
WIC (Mujeres, Infantes y Niños)	○	○	○
Asistencia Social	○	○	○
Beneficios de desempleo	○	○	○

Solo unas cuantas preguntas sobre su situación económica

Durante los últimos 12 meses, ¿ha tenido dificultad para pagar lo siguiente?

	No	Sí	Prefiero no contestar esta pregunta
Transportación	◯	◯	◯
Vivienda	◯	◯	◯
Cuidado médico o medicamentos	◯	◯	◯
Comida	◯	◯	◯

Durante el último mes, ¿con qué frecuencia diría usted que ha tenido dinero suficiente para satisfacer sus necesidades básicas como la alimentación, vivienda y transporte?

- ◯ Todo el tiempo
- ◯ La mayor parte del tiempo
- ◯ Alguna parte del tiempo
- ◯ Rara vez
- ◯ Nunca
- ◯ No lo sé
- ◯ Prefiero no contestar esta pregunta

Ahora queremos hacerle algunas preguntas acerca del historial de sus embarazos y los resultados de sus embarazos.

¿Ha estado usted embarazada anteriormente?

◯ No
◯ Sí
◯ Prefiero no contestar esta pregunta

¿Cuántas veces en su vida ha estado embarazada, (independientemente del resultado)?

◯ 0
◯ 1
◯ 2
◯ 3
◯ 4
◯ 5
◯ 6
◯ 7
◯ 8
◯ 9
◯ 10+

¿Cuántas veces en su vida ha tenido partos con nacidos vivos?

◯ 0
◯ 1
◯ 2
◯ 3
◯ 4
◯ 5
◯ 6
◯ 7
◯ 8
◯ 9
◯ 10+

¿Cuántas veces en su vida ha tenido abortos espontáneos?

◯ 0
◯ 1
◯ 2
◯ 3
◯ 4
◯ 5
◯ 6
◯ 7
◯ 8
◯ 9
◯ 10+

¿Cuántas veces en su vida ha tenido abortos provocados?

◯ 0
◯ 1
◯ 2
◯ 3
◯ 4
◯ 5
◯ 6
◯ 7
◯ 8
◯ 9
◯ 10+

¿Cuántas veces en su vida ha tenido partos con nacidos muertos?	◯ 0<br>◯ 1<br>◯ 2<br>◯ 3<br>◯ 4<br>◯ 5<br>◯ 6<br>◯ 7<br>◯ 8<br>◯ 9<br>◯ 10+
¿Cuántas veces en su vida ha tenido embarazos ectópicos (en las trompas)?	◯ 0<br>◯ 1<br>◯ 2<br>◯ 3<br>◯ 4<br>◯ 5<br>◯ 6<br>◯ 7<br>◯ 8<br>◯ 9<br>◯ 10+
¿Cuántas veces en su vida ha dado a su recién nacido en adopción?	◯ 0<br>◯ 1<br>◯ 2<br>◯ 3<br>◯ 4<br>◯ 5<br>◯ 6<br>◯ 7<br>◯ 8<br>◯ 9<br>◯ 10+
¿Cuántas veces en su vida ha quedado embarazada cuando no lo quería estar?	◯ 0<br>◯ 1<br>◯ 2<br>◯ 3<br>◯ 4<br>◯ 5<br>◯ 6<br>◯ 7<br>◯ 8<br>◯ 9<br>◯ 10+
¿Qué edad tenia usted cuando quedó embarazada por PRIMERA vez?	______________________
¿Cuándo terminó su ÚLTIMO embarazo? Si usted no puede recordar la fecha exacta, por favor haga su mejor cálculo.	______________________

¿En qué resultó su último embarazo?	◯ Aborto espontáneo<br>◯ Aborto provocado<br>◯ Parto con nacido vivo prematuro (menos de 37 semanas)<br>◯ Parto con nacido vivo (37 semanas o más)<br>◯ Ectópico (en las trompas)<br>◯ Prefiero no contestar esta pregunta

Ahora nos gustaría preguntarle acerca de sus planes de tener criaturas en el futuro, coordinación ideal, y sentimientos sobre el embarazo.

¿Piensa usted que le gustaría tener criaturas (o más criaturas) en el futuro?

- ◯ No
- ◯ Sí,
- ◯ No lo sé

¿Cuáles son sus planes para el embarazo en el futuro?

- ◯ No tengo planes de quedar embarazada en cualquier momento en el futuro
- ◯ Me gustaría quedar embarazada en el próximo año
- ◯ Me gustaría quedar embarazada en los próximos 2-5 años (pero no en el próximo año)
- ◯ Me gustaría quedar embarazada en los próximos 5-10 años , pero no antes de esa fecha .
- ◯ No estoy segura de si o cuando me gustaría quedar embarazada
- ◯ Otros

¿Cuáles son sus planes de embarazo ?

____

¿Qué tan importante es para usted no embarazarse antes de estar lista?

No importante para nada — Lo más importante

(Place a mark on the scale above)

¿Qué tan importante es para usted no embarazarse ahora o en el futuro?

No importante para nada — Lo más importante

(Place a mark on the scale above)

¿Cómo se sentiría si quedara embarazada en el próximo mes?

El peor de sentimientos — Lo más contenta posible

(Place a mark on the scale above)

Por favor comparta un poco más cómo le afectaría la vida un embarazo ahora o en el futuro cercano.

____

Ahora queremos hacerle algunas preguntas acerca de los métodos que ha utilizado para prevenir el embarazado.

¿Cuáles son TODOS los métodos anticonceptivos que usted ha usado en el pasado para prevenir un embarazo?

- ☐ Implante anticonceptivo (Nexplanon)
- ☐ DIU sin hormonas de cobre (Paragard)
- ☐ DIU hormonal de 5 años (Mirena)
- ☐ DIU hormonal de 3 años (Liletta)
- ☐ Otro DIU hormonal de 3 años (Skyla)
- ☐ Inyección de 3 meses (Depo-Provera)
- ☐ Pastilla anticonceptiva (combinación hormonal)
- ☐ Pastilla anticonceptiva (solo progesterona)
- ☐ Parche anticonceptivo (Xulane)
- ☐ Anillo vaginal (NuvaRing)
- ☐ Condones masculinos
- ☐ Condones femeninos
- ☐ Capuchón vaginal o esponja
- ☐ Espermicida
- ☐ Diafragma
- ☐ Conciencia de fertilidad / Planificación familiar natural/el ritmo
- ☐ Retiro
- ☐ Anticonceptivo de emergencia Levonorgestrel (Plan B/Next Choice)
- ☐ Pastilla anticonceptiva de emergencia Ulipristal EC (Ella)
- ☐ Otro
- ☐ Ninguno; no estoy planeando usar un método anticonceptivo

(Marque todas las respuestas que correspondan)

Si hay otro, por favor especifique:

____

¿Qué métodos anticonceptivos ha usado usted en las últimas 4 semanas?

Esto no bebería de incluir el método que usted está recibiendo hoy.

- ☐ Implante anticonceptivo (Nexplanon)
- ☐ DIU sin hormonas de cobre (Paragard)
- ☐ DIU hormonal de 5 años (Mirena)
- ☐ DIU hormonal de 3 años (Liletta)
- ☐ Otro DIU hormonal de 3 años (Skyla)
- ☐ Inyección de 3 meses (Depo-Provera)
- ☐ Pastilla anticonceptiva (combinación hormonal)
- ☐ Pastilla anticonceptiva (solo progesterona)
- ☐ Parche anticonceptivo (Xulane)
- ☐ Anillo vaginal (NuvaRing)
- ☐ Condones masculinos
- ☐ Condones femeninos
- ☐ Capuchón vaginal o esponja
- ☐ Espermicida
- ☐ Diafragma
- ☐ Conciencia de fertilidad / Planificación familiar natural/el ritmo
- ☐ Retiro
- ☐ Anticonceptivo de emergencia Levonorgestrel (Plan B/Next Choice)
- ☐ Pastilla anticonceptiva de emergencia Ulipristal EC (Ella)
- ☐ No he tenido relaciones sexuales
- ☐ Otro
- ☐ Ninguno; no estoy planeando usar un método anticonceptivo

(Marque todas las respuestas que correspondan)

Si hay otro, por favor especifique:

____

¿Por cuánto tiempo ha estado usando este método anticonceptivo?<br><br>Si usted está usando más de un método, conteste cualquier método hormonal.	◯ Menos de 3 meses<br>◯ De 3 a 6 meses<br>◯ De 6 meses a un año<br>◯ De 1 a 2 años<br>◯ De 2 a 3 años<br>◯ Más de 3 años<br>◯ No lo sé<br>◯ Prefiero no contestar esta pregunta
¿Por cuántos años ha usado este método anticonceptivo?	__________________________________
Por lo general, ¿Qué tan satisfecha está usted con el método que estaba usando durante las 4 semanas anteriores?	◯ Completamente satisfecha<br>◯ Algo satisfecha<br>◯ Ni satisfecha o insatisfecha<br>◯ Algo insatisfecha<br>◯ Completamente insatisfecha<br>◯ Prefiero no contestar esta pregunta
Por lo general, ¿qué tanta confianza le tiene al método que ha estado utilizando las últimas 4 semanas para prevenir un embarazo?	◯ Muchísima confianza<br>◯ Mucha confianza<br>◯ Algo de confianza<br>◯ Poca confianza<br>◯ Muy poca o ninguna confianza<br>◯ Prefiero no contestar esta pregunta
Por favor marque si usted está de acuerdo o en desacuerdo con la siguiente frase: "Yo siento que tengo control si quedo o no embarazada".	◯ Estoy muy de acuerdo<br>◯ Estoy un poco de acuerdo<br>◯ No estoy de acuerdo o desacuerdo<br>◯ Estoy un poco en desacuerdo<br>◯ Estoy muy en desacuerdo<br>◯ Prefiero no contestar esta pregunta
Antes de hoy día, ¿había escuchado sobre el dispositivo intrauterino (DIU)?	◯ Yes<br>◯ No
¿Cuál de los siguientes mejor describe su nivel de interés en recibir un dispositivo anticonceptivo hoy día?	◯ No interesada para nada<br>◯ Algo interesada<br>◯ Sumamente interesada
Si usted podría recibir un dispositivo (DIU) hoy día por gratis, ¿le gustaría uno?	◯ No<br>◯ Sí<br>◯ No estoy segura
¿Quería usted un dispositivo (DIU), pero no pudo recibirlo hoy día?	◯ No<br>◯ Sí<br>◯ No estoy segura
Si respondió Sí, por favor explique:	__________________________________
Antes de hoy día, ¿había escuchado sobre el implante anticonceptivo, Nexplanon?	◯ Yes<br>◯ No

¿Cuál de los siguientes mejor describe su nivel de interés en recibir un implante anticonceptivo hoy día?	◯ No interesada para nada<br>◯ Algo interesada<br>◯ Sumamente interesada
Si usted podría recibir un implante anticonceptivo hoy día por gratis, ¿le gustaría uno?	◯ No<br>◯ Sí<br>◯ No estoy segura
¿Quería usted un implante anticonceptivo, pero no pudo recibirlo hoy día?	◯ No<br>◯ Sí<br>◯ No estoy segura
Si respondió Sí, por favor explique:	__________________________________

¿Qué tan importante es cada una de las siguientes características para usted al decidir qué método anticonceptivo usar?

	No importa para nada	Un poco importante	Muy importante	Extremadamente importante	No lo sé/prefiero no contestar esta pregunta
Que no tenga hormonas	○	○	○	○	○
Que mi pareja lo apruebe	○	○	○	○	○
Que no interrumpa las relaciones sexuales	○	○	○	○	○
Que no reduzca mi deseo de tener relaciones sexuales (libido)	○	○	○	○	○
Que no vaya en contra de mis creencias religiosas	○	○	○	○	○
Que sea recomendado por mis amigos (as)	○	○	○	○	○
Que sea el método más efectivo	○	○	○	○	○

Ahora queremos hacerle algunas preguntas acerca de ciertos aspectos de su salud y bienestar que puedan estar relacionados con su ciclo menstrual.

¿En las últimas 4 semanas, algunos de los siguientes síntomas han sido problemas para usted?

	No he tenido este problema en los últimos 30 días	Una vez al mes	Un par de días al mes	Una vez por semana	Un par de días por semana	A diario	No lo sé/prefiero no contestar esta pregunta
Dolores de cabeza	○	○	○	○	○	○	○
Hinchazón del abdomen	○	○	○	○	○	○	○
Sensibilidad en los senos	○	○	○	○	○	○	○
Mal humor o irritabilidad	○	○	○	○	○	○	○
Acné	○	○	○	○	○	○	○
Dolor menstrual	○	○	○	○	○	○	○
Aumento de peso	○	○	○	○	○	○	○
Pérdida de peso	○	○	○	○	○	○	○
Depresión	○	○	○	○	○	○	○
Estreñimiento o diarrea	○	○	○	○	○	○	○

¿Ha buscado usted atención médica a causa de estos problemas?

○ No
○ Sí
○ Prefiero no contestar esta pregunta

Si respondió Sí, por favor de especificar cuál(es) fueron:

____

¿Le recetaron un medicamento para tratar estos problemas?

○ No
○ Sí

A continuación, queremos obtener información acerca de sus relaciones sexuales. Por favor, recuerde que todo lo que nos dices es confidencial.

En una escala del 1 al 100, ¿cómo podría clasificar su vida sexual en este momento?

Lo peor posible — Lo mejor posible

(Place a mark on the scale above)

En las últimas 4 semanas, usted diría que su método anticonceptivo o método para prevenir el embarazo ha:

- ◯ Ha mejorado mi vida sexual mucho
- ◯ Ha mejorado mi vida sexual un poco
- ◯ No ha tenido ningún efecto en mi vida sexual
- ◯ Ha empeorado mi vida sexual un poco
- ◯ Ha empeorado mi vida sexual mucho

Por favor explique brevemente el impacto que el método anticonceptivo que está usando actualmente ha tenido en su vida sexual.

____

¿Ha estado activa sexualmente con un hombre en las últimas 4 semanas? Esto pudiera incluir una variedad de actividades, y no solamente sexo vaginal.

- ◯ No
- ◯ Sí
- ◯ Prefiero no contestar esta pregunta

¿Por cuánto tiempo ha estado usted en esta relación?

Nota: Si usted tiene más de una pareja, cuando conteste, piense en la pareja principal que tiene usted

- ◯ Menos de 3 meses
- ◯ De 3 a 6 meses
- ◯ De 6 meses a un año
- ◯ De 1 a 2 años
- ◯ De 2 a 3 años
- ◯ Más de 3 años

Si usted ha estado con su pareja principal más de 3 años, por favor de decirnos cuantos años han sido.

____
(años)

Ahora vamos a hacerle algunas preguntas sobre sus sentimientos y experiencias sexuales durante las últimas cuatro semanas.

Durante las últimas 4 semanas, ¿cómo calificaría su nivel de deseo o interés sexual?	◯ Muy alto<br>◯ Alto<br>◯ Moderado<br>◯ Bajo<br>◯ Muy bajo o casi nada<br>◯ Prefiero no contestar esta pregunta
Durante las últimas 4 semanas, ¿cómo calificaría su excitación sexual durante el acto sexual?	◯ No he tenido relaciones sexuales<br>◯ Muy alto<br>◯ Alto<br>◯ Moderado<br>◯ Bajo<br>◯ Muy bajo o casi nada<br>◯ Prefiero no contestar esta pregunta
Durante las últimas 4 semanas, ¿qué tan seguido usted se puso mojada durante el acto sexual?	◯ No he tenido relaciones sexuales<br>◯ Casi siempre o siempre<br>◯ La mayor parte del tiempo (más de la mitad)<br>◯ A veces (por lo menos la mitad)<br>◯ De vez en cuando (menos que la mitad)<br>◯ Casi nunca o nunca<br>◯ Prefiero no contestar esta pregunta
Durante las últimas 4 semanas, cuándo tuvo relaciones o estímulo sexual , ¿qué tan seguido usted llegó al orgasmo (clímax)?	◯ No he tenido relaciones sexuales<br>◯ Casi siempre o siempre<br>◯ La mayor parte del tiempo (más de la mitad)<br>◯ A veces (por lo menos la mitad)<br>◯ De vez en cuando (menos que la mitad)<br>◯ Casi nunca o nunca<br>◯ Prefiero no contestar esta pregunta
Durante las últimas 4 semanas por lo general, ¿qué tan satisfecha ha estado con su vida sexual?	◯ Muy satisfecha<br>◯ Moderadamente satisfecha<br>◯ Igualmente satisfecha que insatisfecha<br>◯ Moderadamente insatisfecha<br>◯ Muy insatisfecha<br>◯ Prefiero no contestar esta pregunta
Durante las últimas 4 semanas, ¿usted tuvo incomodidad o dolor durante la penetración vaginal?	◯ No intente de tener relaciones sexuales<br>◯ Casi siempre o siempre<br>◯ La mayor parte del tiempo (más de la mitad)<br>◯ A veces (por lo menos la mitad)<br>◯ De vez en cuando (menos de la mitad)<br>◯ Casi nunca o nunca<br>◯ Prefiero no contestar esta pregunta

Los siguientes puntos se relacionan con sus experiencias sexuales. Usted puede encontrar algunos de estos puntos similares a los que acaba de completar. Eso está bien; haga su mejor esfuerzo para responder a todas las preguntas.

Al responder a estos puntos, por favor piense en las últimas 4 semanas.

Pensando en el último mes, ¿Qué tan satisfecha o insatisfecha ha estado usted con cada uno de los siguientes temas?

	Nada satisfecha	Un poco satisfecha	Moderadamen te satisfecha	Muy satisfecha	Sumamente satisfecha	Prefiero no contestar esta pregunta
La intensidad de mi excitación sexual	○	○	○	○	○	○
La calidad de mis orgasmos	○	○	○	○	○	○
Mi habilidad de entregarme al placer sexual durante el acto sexual	○	○	○	○	○	○
Mi habilidad de enfocarme y concentrarme durante el acto sexual	○	○	○	○	○	○
La manera que yo reacciono sexualmente hacia mi pareja	○	○	○	○	○	○
El funcionamiento de mi cuerpo sexualmente	○	○	○	○	○	○
Mi apertura emocional durante el sexo	○	○	○	○	○	○
Mi estado de ánimo después del acto sexual	○	○	○	○	○	○
La frecuencia de mis orgasmos	○	○	○	○	○	○
El placer que yo le doy a mi pareja	○	○	○	○	○	○
El balance entre lo que yo doy y lo que recibo durante el sexo	○	○	○	○	○	○
La apertura emocional de mi pareja durante el sexo	○	○	○	○	○	○
La motivación que tiene mi pareja para iniciar el sexo	○	○	○	○	○	○
La habilidad que tiene mi pareja de llegar al orgasmo	○	○	○	○	○	○
La habilidad que tiene mi pareja de entregarse al placer sexual	○	○	○	○	○	○

La forma en que mi pareja se encarga de mis necesidades sexuales	◯	◯	◯	◯	◯	◯
La creatividad sexual de mi pareja	◯	◯	◯	◯	◯	◯
La disponibilidad sexual de mi pareja	◯	◯	◯	◯	◯	◯
La variedad de mis actividades sexuales	◯	◯	◯	◯	◯	◯
La frecuencia de mi actividad sexual	◯	◯	◯	◯	◯	◯

¿Tiene usted alguna preocupación acerca de su funcionamiento sexual ?

◯ No
◯ Sí
◯ Prefiero no contestar esta pregunta

Por favor, describa

____

Los últimos puntos tienen que ver más que nada con su bienestar y salud en general.

	Todo el tiempo	La mayor parte del tiempo	Más de la mitad del tiempo	Menos de la mitad del tiempo	A veces	Nunca
Me he sentido alegre y de buen humor	◯	◯	◯	◯	◯	◯
Me he sentido calmada y relajada	◯	◯	◯	◯	◯	◯
Me he sentido llena de ánimo y de energía	◯	◯	◯	◯	◯	◯
Me he despertado fresca y descansada	◯	◯	◯	◯	◯	◯
Mi vida cotidiana ha estado llena de cosas que me interesan	◯	◯	◯	◯	◯	◯

¿Estaría dispuesta a que el personal de este estudio le contactaran para otros estudios en el futuro?

◯ Yes
◯ No

¿Hay otra cosa que deberíamos saber?

____

Cuestionario 1, 3 & 6 Meses

�Bienvenida! Gracias de nuevo por su participaci�n. Nos gustar�a hacer seguimiento con usted sobre sus experiencias desde que inicio en este estudio. Estos resultados podr�an ayudar a mejorar la calidad de la salud reproductiva.

Calculamos que esta encuesta le tomar� 15-30 minutos para completarla. Cuando la termine, usted recibir� un cr�dito hacia una tarjeta de regalo de $20 que recibir� despu�s de terminar la encuesta de los 6 meses.

Haga lo mejor que pueda al contestar cada pregunta, y por favor recuerde que toda esta informaci�n es completamente confidencial.

Le agradecemos calurosamente por su participaci�n.

Muchas gracias por participar en este estudio.

Primero que nada, queremos asegurarnos de que no ha cambiado su información de contacto.

¿Ha cambiado alguno de sus datos de contacto (por ejemplo, su número de teléfono, correo electrónico o dirección)?

- ◯ No
- ◯ Sí

Si es así, ¿cuál es la información que ha cambiado?

- ☐ Nuevo número de teléfono
- ☐ Nuevo correo electrónico
- ☐ Nueva dirección

(Marque todas las respuestas que correspondan)

Por favor de darnos su nuevo número de teléfono.

____
(xxx-xxx-xxxx)

Por favor de darnos su nuevo correo electrónico.

____

Por favor de darnos su nueva dirección.

____

Vamos a empezar por hacerle algunas preguntas sobre el método anticonceptivo que seleccionó al principio del estudio.

¿Qué método(s) anticonceptivos ha usado en las últimas 4 semanas?

- ☐ Implante anticonceptivo (Nexplanon)
- ☐ DIU sin hormonas de cobre (Paragard)
- ☐ DIU hormonal de 5 años (Mirena)
- ☐ DIU hormonal de 3 años (Liletta)
- ☐ Otro DIU hormonal de 3 años (Skyla)
- ☐ Inyección de 3 meses (Depo-Provera)
- ☐ Pastilla anticonceptiva (combinación hormonal)
- ☐ Pastilla anticonceptiva (solo progesterona)
- ☐ Parche anticonceptivo (Xulane)
- ☐ Anillo vaginal (NuvaRing)
- ☐ Condones masculinos
- ☐ Condones femeninos
- ☐ Capuchón vaginal o esponja
- ☐ Espermicida
- ☐ Diafragma
- ☐ Conciencia de fertilidad / Planificación familiar natural/el ritmo
- ☐ Retiro
- ☐ Anticonceptivo de emergencia Levonorgestrel (Plan B/Next Choice)
- ☐ Pastilla anticonceptiva de emergencia Ulipristal EC (Ella)
- ☐ Otro
- ☐ Ninguno; no estoy usar un método anticonceptivo

(Marque todas las respuestas que correspondan)

Si hay otro, por favor de especificar: ________________________________

¿Sigue usando el el mismo método anticonceptivo que recibió al principio del estudio?

- ◯ No
- ◯ Sí

En las últimas 4 semanas, ¿ha comprobado para asegurarse que su DIU o implante todavía sigue en su lugar?

- ◯ No, no lo he hecho
- ◯ Sí, yo he tocado los hilos (o barra en el brazo) y confirmé que sigue ahí
- ◯ Sí, un proveedor médico confirmó los hilos
- ◯ Intenté, pero no pude sentir los hilos

¿Por cuánto tiempo piensa usar el método anticonceptivo que está usando?

- ◯ Menos de 1 año
- ◯ Más de 1 año, pero menos de 2 años
- ◯ Más de 2 años, pero menos de 3 años
- ◯ Más de 3 años, pero menos de 5 años
- ◯ Más de 5 años, pero menos de 10 años
- ◯ Más de 10 años
- ◯ No estoy segura
- ◯ Prefiero no contestar esta pregunta

¿Cuáles son las razones por las que ya no está usando el método anticonceptivo que recibió al inicio del estudio?	☐ Los problemas de sangrado (manchado , irregular, etc.)<br>☐ Demasiado sangrado<br>☐ Dolor menstrual<br>☐ Dolor<br>☐ Problemas en los senos<br>☐ Aumento de peso<br>☐ La pérdida de peso<br>☐ Mal humor o irritabilidad<br>☐ Hinchazón del abdomen<br>☐ Problemas de la piel<br>☐ Dolor durante el sexo<br>☐ Mi pareja podía sentir el DIU<br>☐ Se cayó/fue expulsado el DIU<br>☐ Quería quedar embarazada<br>☐ Tuve una prueba de embarazo positiva<br>☐ Otro<br>(Marque todas las respuestas que correspondan)
Si es otro, por favor especifique:	__________________________________
¿En qué fecha dejó de usar el método anticonceptivo que recibió al incio del estudio?	__________________________________<br>(Por favor calcule si no está segura)
¿Cuándo comenzó a usar el nuevo método?	__________________________________<br>(Por favor calcule si no está segura)
¿Cuáles de las siguientes opciones describe mejor su sangrado vaginal en las últimas 4 semanas?	◯ No he tenido sangrado vaginal<br>◯ He tenido menos sangrado vaginal<br>◯ No he tenido ningún cambio en mi sangrado vaginal<br>◯ He tenido más sangrado vaginal

Las siguientes preguntas son sobre los problemas de salud o de su embarazo que puedan estar relacionados con el anticonceptivo desde que empezó el estudio.

En las últimas 4 semanas, ¿se ha hecho una prueba del embarazo?	◯ No<br>◯ Sí, me hice una prueba en la casa<br>◯ Sí, me hicieron una prueba en la clínica<br>◯ No lo sé
¿Cuál fue el resultado de la prueba de embarazo?	◯ Negativo<br>◯ Positivo<br>◯ No lo sé
¿En qué fecha se hizo la prueba?	__________________________________
Cuando quedó embarazada, ¿estaba tratando de quedar embarazada?	◯ Si<br>◯ No

Si a usted le salió la prueba positiva, por favor de indicar el resultado del embarazo.	◯ Tuve o estoy planeando tener un aborto provocado<br>◯ Tuve un aborto espontáneo<br>◯ Tuve un embarazo ectópico<br>◯ Estoy planeando en continuar con el embarazo y criar a mi bebé<br>◯ Estoy planeando en continuar con el embarazo y dar al bebé en adopción<br>◯ No estoy segura de que voy hacer
¿En qué fecha terminó su embarazo, independientemente del resultado? Si usted todavía está embarazada y quiere continuar con el embarazo por favor ponga su fecha probable de parto.	__________________________
Para garantizar su seguridad, si usted estuvo embarazada, nos gustaría saber más sobre qué tipo de atención recibió. Por favor, indique el nombre de la clínica o del hospital donde la atendieron.	________________________________
Desde que empezó el estudio, ¿ha sido hospitalizada debido a una enfermedad o una lesión?	◯ No<br>◯ Sí
Por favor de darnos la fecha en que la hospitalizaron:	__________________________
Por favor de explicarnos que pasó:	________________________________
¿Ha sido atendida por un proveedor médico debido a un problema que usted pensaba que pudiera estar relacionado con el método anticonceptivo?	◯ No<br>◯ Sí
Por favor de darnos la fecha:	__________________________
Por favor de explicarnos el problema y que pasó:	________________________________

Ahora queremos hacerle algunas preguntas sobre diferentes métodos anticonceptivos. Piense en el(los) método(s) que ha usado en las últimas 4 semanas.

Por lo general, ¿Qué tan satisfecha está usted con el método que estaba usando durante las 4 semanas anteriores?	◯ Completamente satisfecha<br>◯ Algo satisfecha<br>◯ Ni satisfecha o insatisfecha<br>◯ Algo insatisfecha<br>◯ Completamente insatisfecha<br>◯ Prefiero no contestar esta pregunta
Por lo general, ¿qué tanta confianza le tiene al método que ha utilizado durante las últimas 4 semanas para prevenir un embarazo?	◯ Muchísima confianza<br>◯ Mucha confianza<br>◯ Algo de confianza<br>◯ Poca confianza<br>◯ Muy poca o ninguna confianza<br>◯ Prefiero no contestar esta pregunta

Por favor marque si usted está de acuerdo o en desacuerdo con la siguiente frase: "Yo siento que tengo el control sobre si o no salgo embarazada."

- ◯ Estoy muy de acuerdo
- ◯ Estoy un poco de acuerdo
- ◯ No estoy de acuerdo o en desacuerdo
- ◯ Estoy un poco en desacuerdo
- ◯ Estoy muy en desacuerdo
- ◯ Prefiero no contestar esta pregunta

Ahora queremos hacerle algunas preguntas acerca de ciertos aspectos de su salud y bienestar que pudieran estar relacionados con su ciclo menstrual.

En las últimas 4 semanas, ¿han sido un problema para usted los siguientes asuntos de salud?

	No he tenido este problema en los últimos 30 días	Una vez al mes	Un par de días al mes	Una vez por semana	Un par de días por semana	A diario	No lo sé
Dolores de cabeza	◯	◯	◯	◯	◯	◯	◯
Hinchazón del abdomen	◯	◯	◯	◯	◯	◯	◯
Sensibilidad en los senos	◯	◯	◯	◯	◯	◯	◯
Mal humor o irritabilidad	◯	◯	◯	◯	◯	◯	◯
Acné	◯	◯	◯	◯	◯	◯	◯
Dolor abdominal	◯	◯	◯	◯	◯	◯	◯
Aumento de peso	◯	◯	◯	◯	◯	◯	◯
Pérdida de peso	◯	◯	◯	◯	◯	◯	◯
Depresión	◯	◯	◯	◯	◯	◯	◯
Estreñimiento o diarrea	◯	◯	◯	◯	◯	◯	◯

¿Ha buscado usted atención médica a causa de estos problemas?

- ◯ No
- ◯ Sí
- ◯ No lo sé

Si es así, por favor de especificar cuáles fueron:

____

¿Le recetaron un medicamento para tratar algunos de estos problemas?

- ◯ No
- ◯ Sí

A continuación, queremos obtener información sobre sus relaciones sexuales. Por favor, recuerde que todo lo que nos dice es confidencial.

Por favor escoja la descripción que más ajusta a lo que usted siente de sí misma.

- ◯ Exclusivamente heterosexual
- ◯ Mayormente heterosexual
- ◯ Bisexual - atraída a hombres y mujeres igualmente
- ◯ Mayormente gay/lesbiana
- ◯ No atraída ni a hombres ni mujeres
- ◯ No sé
- ◯ Prefiero no contestar

¿Ha tenido relaciones sexuales en las últimas 4 semanas?	◯ No<br>◯ Sí<br>◯ Prefiero no contestar esta pregunta
¿Es esta pareja la misma persona con la cual usted estaba teniendo relaciones sexuales la última vez que usted contestó el cuestionario anterior?	◯ No<br>◯ Sí<br>◯ Prefiero no contestar esta pregunta
¿Cuál es el sexo de su pareja principal durante las últimas 4 semanas?	◯ Varón<br>◯ Hembra<br>◯ Otro<br>◯ No lo sé<br>◯ Prefiero no contestar
En una escala del 1 al 100, ¿cómo usted calificaría su vida sexual en este momento?	Lo peor posible — Lo mejor posible<br>*(Place a mark on the scale above)*
¿Qué es lo que usted piensa que tendría que cambiar para mejorar y llegar al 100?	____________________________
En las últimas 4 semanas, usted diría que su método ha:	◯ Ha mejorado mi vida sexual mucho<br>◯ Ha mejorado mi vida sexual un poco<br>◯ No ha tenido ningún efecto en mi vida sexual<br>◯ Ha empeorado mi vida sexual un poco<br>◯ Ha empeorado mi vida sexual mucho
Por favor de explicar el impacto que su método anticonceptivo ha tenido en su vida sexual.	____________________________
¿Usted tiene alguna preocupación con su funcionamiento sexual?	◯ No<br>◯ Sí<br>◯ No estoy segura
Por favor de describirlo brevemente:	____________________________

Ahora vamos a hacerle algunas preguntas sobre sus sentimientos y experiencias sexuales durante las últimas cuatro semanas. Haga lo mejor que pueda al responder cada pregunta.

Durante las últimas 4 semanas, ¿cómo calificaría su nivel de deseo o interés sexual?	◯ Muy alto<br>◯ Alto<br>◯ Moderado<br>◯ Bajo<br>◯ Muy bajo o casi nada<br>◯ Prefiero no contestar esta pregunta

Durante las últimas 4 semanas, ¿cómo calificaría su excitación durante el acto sexual?	◯ No he tenido relaciones sexuales<br>◯ Muy alto<br>◯ Alto<br>◯ Moderado<br>◯ Bajo<br>◯ Muy bajo o casi nada<br>◯ Prefiero no contestar esta pregunta
Durante las últimas 4 semanas, ¿qué tan seguido usted se puso mojada durante el acto sexual?	◯ No intenté tener relaciones sexuales<br>◯ Casi siempre o siempre<br>◯ La mayor parte del tiempo (más de la mitad)<br>◯ A veces (por lo menos la mitad)<br>◯ De vez en cuando (menos de la mitad)<br>◯ Casi nunca o nunca<br>◯ Prefiero no contestar esta pregunta
Durante las últimas 4 semanas, cuando tuvo relaciones sexuales o solamente estímulo, ¿qué tan seguido usted llegó al orgasmo (clímax)?	◯ No he tenido relaciones sexuales<br>◯ Casi siempre o siempre<br>◯ La mayor parte del tiempo (más de la mitad)<br>◯ A veces (por lo menos la mitad)<br>◯ De vez en cuando (menos de la mitad)<br>◯ Casi nunca o nunca<br>◯ Prefiero no contestar esta pregunta
Durante las últimas 4 semanas por lo general, ¿qué tan satisfecha ha estado con su vida sexual?	◯ Muy satisfecha<br>◯ Moderadamente satisfecha<br>◯ Igualmente satisfecha e insatisfecha<br>◯ Moderadamente insatisfecha<br>◯ Muy insatisfecha<br>◯ Prefiero no contestar esta pregunta
Durante las últimas 4 semanas, ¿qué tan a menudo usted tuvo incomodidad o dolor durante la penetración vaginal?	◯ No intenté tener relaciones sexuales<br>◯ Casi siempre o siempre<br>◯ La mayor parte del tiempo (más de la mitad)<br>◯ A veces (por lo menos la mitad)<br>◯ De vez en cuando (menos de la mitad)<br>◯ Casi nunca o nunca<br>◯ Prefiero no contestar esta pregunta

Los siguientes puntos se relacionan con sus experiencias sexuales. Usted puede encontrar algunos de estos puntos similares a los que acaba de completar. Eso está bien; haga su mejor esfuerzo para responder a todas las preguntas.

Al responder estos puntos, por favor piense en las últimas 4 semanas. Pensando en el último mes, ¿qué tan satisfecha o insatisfecha ha estado usted con cada uno de los siguientes temas?

	Nada satisfecha	Un poco satisfecha	Moderadamente satisfecha	Muy satisfecha	Extremadamente satisfecha	Prefiero no contestar esta pregunta
La intensidad de me excitación sexual	○	○	○	○	○	○
La calidad de mis orgasmos	○	○	○	○	○	○
Mi habilidad de entregarme al placer sexual durante el acto sexual	○	○	○	○	○	○
Mi habilidad de enfocarme y concentrarme durante el acto sexual	○	○	○	○	○	○
La manera que yo reacciono sexualmente hacia mi pareja	○	○	○	○	○	○
El funcionamiento de mi cuerpo sexualmente	○	○	○	○	○	○
Mi apertura emocional durante el acto sexual	○	○	○	○	○	○
Mi estado de ánimo después del acto sexual	○	○	○	○	○	○
La frecuencia de mis orgasmos	○	○	○	○	○	○
El placer que yo le doy a mi pareja	○	○	○	○	○	○
El balance entre lo que yo doy y lo que recibo durante el acto sexual	○	○	○	○	○	○
La apertura emocional de mi pareja durante el acto sexual	○	○	○	○	○	○
La motivación que tiene mi pareja para iniciar el sexo	○	○	○	○	○	○
La habilidad que tiene mi pareja de llegar al orgasmo	○	○	○	○	○	○
La habilidad que tiene mi pareja de entregarse al placer sexual	○	○	○	○	○	○
La forma en que mi pareja se encarga de mis necesidades sexuales	○	○	○	○	○	○

La creatividad sexual de mi pareja	○	○	○	○	○	○
La disponibilidad sexual de mi pareja	○	○	○	○	○	○
La variedad de mis actividades sexuales	○	○	○	○	○	○
La frecuencia de mi actividad sexual	○	○	○	○	○	○

Los últimos puntos tienen que ver más que nada con su bienestar y salud en general. Por favor de pensar como se ha sentido usted en las últimas 4 semanas.

	Siempre	La mayor parte del tiempo	Más de la mitad del tiempo	Menos de la mitad del tiempo	Muy pocas veces	Nunca
Me he sentido alegre y de buen humor	○	○	○	○	○	○
Me he sentido calmada y relajada	○	○	○	○	○	○
Me he sentido llena de ánimo y de energía	○	○	○	○	○	○
Me desperté sintiéndome fresca y descansada	○	○	○	○	○	○
Mi vida cotidiana está llena de cosas que me interesan	○	○	○	○	○	○

Gracias de nuevo por participar en este estudio. En el siguiente espacio, no dude en decirnos cualquier cosa sobre su experiencia con su método anticonceptivo o con su participación en este estudio. De lo contrario, nos pondremos en contacto con usted cuando sea tiempo para la siguiente parte del estudio.

Comentarios: ______________________________

Cuestionario 1, 2 & 3 Años

¡Bienvenida de vuelta! Como ha de recordar, este estudio nos está ayudando a aprender más acerca de la relación entre las experiencias anticonceptivas de las personas. Su contribución es apreciada aun si dejó de usar el método que recibió al inicio o si cambió métodos. Los descubrimientos podrían ayudar a mejorar la calidad de la atención de la salud reproductiva.

Calculamos que esta encuesta le tomará menos de 10 minutos en completarse. Una vez que complete la encuesta, usted recibirá un código de regalo de $ 20 para Amazon.com por medio de su correo electrónico como agradecimiento por su tiempo. Esto puede tomar hasta dos semanas desde la fecha en la que completó la encuesta.

Usted recibirá 3 recordatorios adicionales hasta que haya completada esta encuesta. Conteste lo mejor que pueda y recuerde que toda su información es completamente confidencial. Gracias por su participación.

Muchas gracias por participar en este estudio.

Primero que nada, queremos asegurarnos de que no ha cambiado su información de contacto.

¿En los últimos 6 meses, ha cambiado alguno de sus datos de contacto (por ejemplo, su número de teléfono, correo electrónico o dirección)?	○ No<br>○ Sí
Si es así, ¿cuál es la información que ha cambiado?	☐ Nuevo número de teléfono<br>☐ Nuevo correo electrónico<br>☐ Nueva dirección<br>(Marque todas las respuestas que correspondan)
Por favor de darnos su nuevo número de teléfono.	____________________<br>(xxx-xxx-xxxx)
Por favor de darnos su nuevo correo electrónico.	____________________
Por favor de darnos su nueva dirección.	____________________

Las siguientes preguntas se tratan de sus planes de quedar embarazada y cambios recientes en su método anticonceptivo.

¿Qué método(s) ha usado para no quedar embarazada en las últimas 4 semanas?	☐ Implante anticonceptivo (Nexplanon)<br>☐ DIU sin hormonas de cobre (Paragard)<br>☐ DIU hormonal de 5 años (Mirena)<br>☐ DIU hormonal de 3 años (Liletta)<br>☐ Otro IUD hormonal de 3 años (Skyla)<br>☐ Inyección de 3 meses (Depo-Provera)<br>☐ Pastilla anticonceptiva (combinación hormonal)<br>☐ Pastilla anticonceptiva (solo progesterona)<br>☐ Parche anticonceptivo (Xuzane)<br>☐ Anillo vaginal (NuvaRing)<br>☐ Condones masculinos<br>☐ Condones femeninos<br>☐ Capuchón vaginal o esponja<br>☐ Espermicida<br>☐ Diafragma<br>☐ Conciencia de fertilidad / Planificación familiar natural/el ritmo<br>☐ Retiro<br>☐ Anticonceptivo de emergencia Levonorgestrel (Plan B/Next Choice)<br>☐ Pastilla anticonceptiva de emergencia Ullipristal EC (Ella)<br>☐ Otro<br>☐ Ninguno; no estoy planeando usar un método anticonceptivo<br>(Marque todas las respuestas que correspondan)
Si hay otro, por favor de especificar:	__________________________________
¿Sigue usando el mismo método anticonceptivo que recibió al principio del estudio?	◯ No<br>◯ Sí
En las últimas 4 semanas, ¿ha comprobado para asegurarse que su DIU o implante es todavía en su lugar?	◯ No, no lo he hecho<br>◯ Sí, yo he tocado los hilos (o barra en el brazo) y confirmé que sigue ahí<br>◯ Sí, un proveedor médico confirmó los hilos<br>◯ Intenté, pero no pude sentir los hilos
¿Por cuánto tiempo piensa usar el método anticonceptivo que está usando?	◯ Menos de 1 año<br>◯ Más de 1 año, pero menos de 2 años<br>◯ Más de 2 años, pero menos de 3 años<br>◯ Más de 3 años, pero menos de 5 años<br>◯ Más de 5 años, pero menos de 10 años<br>◯ Más de 10 años<br>◯ No estoy segura<br>◯ Prefiero no contestar esta pregunta

¿Cuáles son las razones por las que ya no está usando el método que comenzó al principio del estudio?	☐ Los problemas de sangrado (manchado, irregular, etc.)<br>☐ Demasiado sangrado<br>☐ Dolor menstrual<br>☐ Dolor<br>☐ Problemas en los senos<br>☐ Aumento de peso<br>☐ La pérdida de peso<br>☐ Mal humor o irritabilidad<br>☐ Hinchazón del abdomen<br>☐ Problemas de la piel<br>☐ Dolor durante el sexo<br>☐ Mi pareja podía sentir el DIU<br>☐ Se cayó/fue expulsado el DIU<br>☐ Quería quedar embarazada<br>☐ Tuve una prueba de embarazo positiva<br>☐ Otro
Si es otro, por favor especifique:	__________________________________
¿En qué fecha le quitaron o se le cayó el aparato?<br><br>Si usted no puede recordar la fecha exacta, haga su mejor cálculo.	__________________________________
¿Cuándo comenzó a usar el nuevo método?	__________________________________<br>((Por favor calcule si no está segura))
Por lo general, ¿qué tan satisfecha ha estado con el método que ha utilizado durante las últimas 4 semanas?	◯ Completamente satisfecha<br>◯ Algo satisfecha<br>◯ Ni satisfecha o insatisfecha<br>◯ Algo insatisfecha<br>◯ Completamente insatisfecha<br>◯ Prefiero no contestar esta pregunta
Por lo general, ¿qué tanta confianza le tiene al método que ha utilizado durante las últimas 4 semanas para prevenir un embarazo?	◯ Muchísima confianza<br>◯ Mucha confianza<br>◯ Algo de confianza<br>◯ Poca confianza<br>◯ Muy poca o ninguna confianza<br>◯ Prefiero no contestar esta pregunta
Por favor marque si usted está acuerdo o en desacuerdo con la siguiente frase: "Yo siento que tengo el control sobre si o no salgo embarazada."	◯ Estoy muy de acuerdo<br>◯ Estoy un poco de acuerdo<br>◯ No estoy de acuerdo o en desacuerdo<br>◯ Estoy un poco en desacuerdo<br>◯ Estoy muy en desacuerdo<br>◯ Prefiero no contestar esta pregunta
En las últimas 4 semanas, usted diría que su método anticonceptivo o método para prevenir el embarazo ha:	◯ Ha mejorado mi vida sexual mucho<br>◯ Ha mejorado mi vida sexual un poco<br>◯ No ha tenido ningún efecto en mi vida sexual<br>◯ Ha empeorado mi vida sexual un poco<br>◯ Ha empeorado mi vida sexual mucho

Por favor explique brevemente el impacto que el método anticonceptivo que está usando actualmente ha tenido en su vida sexual. __________________________________

Ahora nos gustaría preguntarle acerca de sus planes de tener criaturas en el futuro, coordinación ideal, y sus sentimientos sobre el embarazo.

¿Piensa usted que le gustaría tener criaturas (o más criaturas) en el futuro?
- ◯ No
- ◯ Sí
- ◯ No lo sé

¿Cuáles son sus planes para el embarazo en el futuro?
- ◯ No tengo planes de quedar embarazada en cualquier momento en el futuro
- ◯ Actualmente, estoy intentando quedar embarazada
- ◯ Me gustaría quedar embarazada en el próximo año
- ◯ Me gustaría quedar embarazada en los próximos 2-5 años (pero no este año)
- ◯ Me gustaría quedar embarazada en los próximos 5-10 años, pero no antes de esa fecha.
- ◯ No lo sé
- ◯ Otro

¿Cuales son sus planes para el embarazo? ____________________________

¿Qué tan importante es para usted no embarazarse antes de estar lista?

No importante para nada — Lo más importante

(Place a mark on the scale above)

¿Qué tan importante es para usted no embarazarse ahora o en el futuro?

No importante para nada — Lo más importante

(Place a mark on the scale above)

¿Cómo se sentiría si se quedara embarazada en el próximo mes?

El peor de sentimientos — Lo más contenta posible

(Place a mark on the scale above)

Por favor comparta un poco más cómo le afectaría la vida un embarazo ahora o en el futuro cercano. __________________________________

¿Qué método(s) está usando actualmente?	☐ Nexplanon<br>☐ DIU sin hormonas de cobre (Paragard)<br>☐ DIU hormonal de 5 años (Mirena)<br>☐ DIU hormonal de 3 años (Liletta)<br>☐ Otro DIU hormonal de 3 años (Skyla)<br>☐ Inyección de 3 meses (Depo-Provera)<br>☐ Pastilla anticonceptiva (combinación hormonal)<br>☐ Pastilla anticonceptiva (solo progesterona)<br>☐ Parche anticonceptivo (Xulane)<br>☐ Anillo vaginal (NuvaRing)<br>☐ Condones masculinos<br>☐ Condones femeninos<br>☐ Capuchón vaginal o esponja<br>☐ Espermicida<br>☐ Diafragma<br>☐ Conciencia de fertilidad / Planificación familiar natural/el ritmo<br>☐ Retiro<br>☐ Anticonceptivo de emergencia (Plan B/Next Choice)<br>☐ Pastilla anticonceptiva de emergencia Ulipristal EC (Ella)<br>☐ Otro<br>☐ Ninguno; no estoy usando nigún método anticonceptivo

Por favor díganos más sobre usted. Ninguna de estas preguntas afectará los servicios que usted recibe.

Reconocemos que no todas las personas que quieran o necesiten anticoncepción se identifican como mujeres.<br><br>Para la siguiente pregunta, por favor seleccione las palabras que usted utiliza para describirse personalmente, o escriba en sus propias palabras como se identifica.	☐ Mujer<br>☐ Hombre<br>☐ No binario<br>☐ Transgénero<br>☐ Prefiero auto-describirme
Escriba en sus propias palabras como se identifica.	__________________________________
Por favor escoja la descripción sobre su identidad sexual que más ajusta a lo que usted siente de sí misma.	◯ Exclusivamente heterosexual<br>◯ Mayormente heterosexual<br>◯ Bisexual - atraída a hombres y mujeres igualmente<br>◯ Mayormente gay/lesbiana<br>◯ No atraída ni a hombres ni mujeres<br>◯ No sé<br>◯ Prefiero no contestar
En los últimos 12 meses, ha tenido sexo con:	◯ Actualmente no activa sexualmente<br>◯ Solo hombres<br>◯ Hombres y mujeres<br>◯ Solo mujeres<br>◯ Prefiero no contestar

¿De las siguientes opciones, qué es lo que mejor describe su estado civil en este momento?	◯ Casada<br>◯ Soltera, pero vivo con mi pareja o en una relación de compromiso<br>◯ Salgo en citas, pero NO en una relación de compromiso<br>◯ Divorciada/Separada<br>◯ Soltera (sin ninguna relación)<br>◯ Viuda<br>◯ Otro<br>◯ Prefiero no contestar esta pregunta
¿Qué tipo de seguro médico tiene usted en este momento?	◯ Ninguno<br>◯ Medicaid<br>◯ Seguro médico del trabajo o que se compra por propia cuenta<br>◯ Seguro médico de estudiante<br>◯ Seguro por parte de mis padres<br>◯ Militar o Veteranos (Champus, ChampVA, Tricare)<br>◯ Invalidez o Medicare<br>◯ No lo sé<br>◯ Prefiero no contestar esta pregunta
¿Qué es lo que mejor describe su situación de empleo actual?	☐ Desempleada<br>☐ Empleada de tiempo completo (mínimo 30 horas/semana)<br>☐ Empleada de medio tiempo (menos de 30 horas/semana<br>☐ Invalidez o en permiso por enfermedad<br>☐ Jubilada<br>☐ Ama de casa<br>☐ Estudiante<br>☐ Otro<br>☐ Prefiero no contestar esta pregunta
Si su situación de empleo es diferente, por favor de describirlo:	__________________________________
¿Cuantas horas trabaja a la semana?	__________________________________<br>(Su mejor cálculo está bien)
¿Cuánto le pagan por hora?	__________________________________
¿Qué tipo de trabajo hace?	____
¿En este momento está buscando trabajo, ya sea trabajo adicional o un diferente trabajo?	◯ No<br>◯ Sí<br>◯ No lo sé<br>◯ Prefiero no contestar esta pregunta
¿Fue usted encarcelada en la cárcel o prisión en los últimos 12 meses? (aun si sólo fue por algunas horas o días)	◯ No<br>◯ Sí<br>◯ Prefiero no contestar esta pregunta

¿Cuantas veces fue usted encarcelada en los últimos 12 meses?

- ◯ 1
- ◯ 2
- ◯ 3
- ◯ 4
- ◯ 5
- ◯ 6
- ◯ 7
- ◯ 8
- ◯ 9
- ◯ 10
- ◯ 11
- ◯ 12 o más

Por favor enumere las fechas en que fue encarcelada y en donde (su mejor cálculo está bien)

Por ejemplo:
1) 1/15/16 - 1/20/16, La cárcel del condado de Salt Lake
2) 2/5/16 - 4/5/16, La prisión del estado de Utah

____

¿Pudo continuar usando anticonceptivos mientras estaba encarcelada?

- ◯ Yes
- ◯ No

Por favor describa las formas en que su encarcelamiento le impacto el uso de anticonceptivos o planes de embarazo.

____

Las siguientes preguntas son sobre su nivel de educación.

¿Qué es lo que mejor describe el nivel más alto de educación que usted ha TERMINADO hasta ahora?

- ◯ Grado 11 o menos
- ◯ Grado 12 (terminé el bachillerato o GED)
- ◯ Educación vocacional/técnica
- ◯ Título de 2 años o algo de Universidad
- ◯ Título de universidad (4 años)
- ◯ Título posgrado (maestría, doctorado, JD, MD, etc.)
- ◯ No lo sé
- ◯ Prefiero no contestar esta pregunta

¿Está usted actualmente en la escuela, ya sea a tiempo completo o medio tiempo?

- ◯ No en este momento
- ◯ Medio tiempo
- ◯ Tiempo completo
- ◯ Prefiero no contestar esta pregunta

¿Qué tipo de título quisiera obtener?

- ◯ Tengo planes de terminar el bachillerato o GED
- ◯ Pienso obtener una educación vocacional/técnica
- ◯ Obtener un título de 2 años
- ◯ Obtener un título de universidad (4 años)
- ◯ Obtener una educación de posgrado (maestría, doctorado, JD, MD, etc.)
- ◯ No lo sé
- ◯ Prefiero no contestar esta pregunta

Por favor de especificar qué tipo:

____

¿En qué fecha espera graduarse?

____

¿Qué es lo que mejor describe sus PLANES para completar el nivel más alto de educación que usted quisiera obtener en el futuro?

- ◯ Ninguno, ya termine la escuela y no tengo planes de regresar
- ◯ Tengo planes de terminar el bachillerato o GED
- ◯ Pienso obtener una educación vocacional/técnica
- ◯ Obtener un título de 2 años
- ◯ Obtener un título de universidad (4 años)
- ◯ Obtener una educación de posgrado (maestría, doctorado, JD, MD, etc.)
- ◯ No lo sé
- ◯ Prefiero no contestar esta pregunta

Sólo unas pocas preguntas más sobre su situación financiera.

¿Cuál es el ingreso anual de su familia?

- ◯ Menos de $10,000
- ◯ $10,000-$19,999
- ◯ $20,000-$29,999
- ◯ $30,000-$39,999
- ◯ $40,000-$49,999
- ◯ $50,000-$59,999
- ◯ $60,000-$69,999
- ◯ $70,000-$79,999
- ◯ $80,000 o más
- ◯ No lo sé
- ◯ Prefiero no contestar esta pregunta

¿Cuál es el mejor cálculo del ingreso anual en su hogar?

____

¿Cuántas personas viven en su hogar?

- ◯ 1
- ◯ 2
- ◯ 3
- ◯ 4
- ◯ 5
- ◯ 6
- ◯ 7
- ◯ 8
- ◯ 9
- ◯ 10
- ◯ 11
- ◯ 12
- ◯ 13
- ◯ 14
- ◯ 15+

¿Cuántos niños menores de 18 años viven con usted en su casa?

____

Por favor de marcar de las siguientes opciones que han sido fuentes de ingreso en el último mes:

- ☐ Yo sola
- ☐ Mi esposo/pareja
- ☐ Otro miembro de la familia/ familiares
- ☐ Asistencia del gobierno
- ☐ Otro
- ☐ Prefiero no contestar esta pregunta

¿Cuáles fueron otras fuentes de ingreso en el último mes?

____

¿Cuánto dinero ganó durante el mes pasado?

- ◯ Nada
- ◯ $1-$400
- ◯ $401-$800
- ◯ $801-$1,200
- ◯ $1,201-$1,600
- ◯ $1,601-$2,000
- ◯ $2,001-$2,400
- ◯ $2,401-$2,800
- ◯ Más de $2,800
- ◯ No lo sé
- ◯ Prefiero no contestar esta pregunta

¿Cuánto dinero ganó su pareja o esposo durante el mes pasado?

- ◯ Nada
- ◯ $1-$400
- ◯ $401-$800
- ◯ $801-$1,200
- ◯ $1,201-$1,600
- ◯ $1,601-$2,000
- ◯ $2,001-$2,400
- ◯ $2,401-$2,800
- ◯ Más de $2,800
- ◯ No lo sé
- ◯ Prefiero no contestar esta pregunta

¿Cuánto dinero contribuyeron otros miembros de su familia/ familiares para el ingreso familiar durante el mes pasado?

- ◯ Nada
- ◯ $1-$400
- ◯ $401-$800
- ◯ $801-$1,200
- ◯ $1,201-$1,600
- ◯ $1,601-$2,000
- ◯ $2,001-$2,400
- ◯ $2,401-$2,800
- ◯ Más de $2,800
- ◯ No lo sé
- ◯ Prefiero no contestar esta pregunta

¿Cuánto dinero recibió por parte de asistencia del gobierno para su ingreso familiar durante el mes pasado?

- ◯ Nada
- ◯ $1-$400
- ◯ $401-$800
- ◯ $801-$1,200
- ◯ $1,201-$1,600
- ◯ $1,601-$2,000
- ◯ $2,001-$2,400
- ◯ $2,401-$2,800
- ◯ Más de $2,800
- ◯ No lo sé
- ◯ Prefiero no contestar esta pregunta

¿Cuánto dinero recibió de otros recursos durante el mes pasado?

- ◯ Nada
- ◯ $1-$400
- ◯ $401-$800
- ◯ $801-$1,200
- ◯ $1,201-$1,600
- ◯ $1,601-$2,000
- ◯ $2,001-$2,400
- ◯ $2,401-$2,800
- ◯ Más de $2,800
- ◯ No lo sé
- ◯ Prefiero no contestar esta pregunta

¿Debió de haber recibido manutención de menores en las últimas 4 semanas?	◯ No<br>◯ Sí<br>◯ Prefiero no contestar esta pregunta
¿Cuánto dinero debió de haber recibido?	__________________________
¿Recibió dinero para manutención de menores durante las últimas 4 semanas?	◯ No<br>◯ Sí<br>◯ Prefiero no contestar esta pregunta
¿Cuánto dinero recibió para manutención de menores durante las últimas 4 semanas?	__________________________
¿Cuál de las siguientes opciones describe mejor su situación de vivienda actual?	◯ Sin vivienda (homeless)<br>◯ Refugio (shelter)<br>◯ Casa rodante (trailer)<br>◯ Apartamento<br>◯ Casa (solo una familia)<br>◯ Otro<br>◯ Prefiero no responder a esta pregunta
Describa que tipo de vivienda:	__________________________

Ahora vamos a hacerle algunas preguntas sobre la asistencia pública que usted puede recibir:

Actualmente usted recibe:

	No	Sí	Prefiero no contestar esta pregunta
Estampillas de comida	◯	◯	◯
WIC (Mujeres, Infantes y Niños)	◯	◯	◯
Asistencia Social	◯	◯	◯
Beneficios de desempleo	◯	◯	◯

Solo unas cuantas preguntas sobre su situación económica

Durante los últimos 12 meses, ¿ha tenido dificultad para pagar lo siguiente?

	No	Sí	Prefiero no contestar esta pregunta
Transportación	◯	◯	◯
Vivienda	◯	◯	◯
Cuidado médico o medicamentos	◯	◯	◯
Comida	◯	◯	◯

Durante el último mes, ¿con qué frecuencia diría usted que ha tenido dinero suficiente para satisfacer sus necesidades básicas como la alimentación, vivienda y transporte?

- ◯ Todo el tiempo
- ◯ La mayor parte del tiempo
- ◯ Alguna parte del tiempo
- ◯ Rara vez
- ◯ Nunca
- ◯ No lo sé
- ◯ Prefiero no contestar esta pregunta

Los últimos puntos tienen que ver con más que nada con su bienestar y salud en general. Por favor piense en las últimas 4 semanas mientras está contestando estas preguntas.

	Todo el tiempo	La mayor parte del tiempo	Más de la mitad del tiempo	Menos de la mitad del tiempo	A veces	Nunca
Me he sentido alegre y de buen humor	◯	◯	◯	◯	◯	◯
Me he sentido calmada y relajada	◯	◯	◯	◯	◯	◯
Me he sentido con llena de ánimo y de energía	◯	◯	◯	◯	◯	◯
Me desperté fresca y descansada	◯	◯	◯	◯	◯	◯
Mi vida cotidiana está llena de cosas que me interesan	◯	◯	◯	◯	◯	◯

Las últimas preguntas son sobre los problemas de salud que pudieran estar relacionados con su método anticonceptivo.

¿Ha tenido una prueba de embarazo positiva desde que contestó el último cuestionario?

- ◯ No
- ◯ Yes
- ◯ No lo sé

¿Cuál fue la fecha de la prueba de embarazo positiva?

____

Cuando quedó embarazada, ¿estaba tratando de quedar embarazada?

- ◯ Si
- ◯ No

Si a usted le salió la prueba positiva, por favor de indicar el resultado del embarazo.

- ◯ Tuve o estoy planeando tener un aborto provocado
- ◯ Tuve un aborto espontáneo
- ◯ Tuve un embarazo ectópico
- ◯ Estoy planeando en continuar con el embarazo y criar a mi bebé
- ◯ Estoy planeando en continuar con el embarazo y dar al bebé en adopción
- ◯ No estoy segura de que voy hacer
- ◯ No he tenido una prueba positiva

¿En qué fecha terminó su embarazo, independientemente del resultado?

____

Para garantizar su seguridad, si usted estuvo embarazada, nos gustaría saber más qué tipo de tención recibió. Por favor, indique el nombre de la clínica o del hospital donde la atendieron.

____

Desde que empezó el estudio, ¿ha sido hospitalizada debido a una enfermedad o una lesión?	◯ No<br>◯ Sí
Por favor de darnos la fecha:	__________________________________
Por favor de explicarnos que pasó:	____
¿Ha sido atendida por un proveedor médico debido a un problema que usted pensaba que pudiera estar relacionado con el DIU o implante que le pusimos?	◯ No<br>◯ Sí
Por favor de darnos la fecha:	__________________________________
Por favor de explicarnos el problema y que pasó:	____

Gracias de nuevo por participar en este estudio.

No dude en decirnos cualquier cosa sobre su experiencia con su método anticonceptivo o con su participación en este estudio

Comentarios:	____

Cuestionario 18 & 30 Meses

Bienvenida de vuelta! Como ha de recordar, este estudio nos está ayudando a aprender más acerca de la relación entre las experiencias anticonceptivas de las personas. Su contribución es apreciada aun si dejó de usar el método que recibió al inicio o si cambió métodos. Los descubrimientos podrían ayudar a mejorar la calidad de la atención de la salud reproductiva.

Usted recibirá 3 recordatorios adicionales hasta que haya completada esta encuesta. Conteste lo mejor que pueda y recuerde que toda su información es completamente confidencial. Gracias por su participación.

Muchas gracias por participar en este estudio. Primero que nada, queremos asegurarnos de que no ha cambiado su información de contacto.

¿En los últimos 6 meses, ha cambiado alguno de sus datos de contacto (por ejemplo, su número de teléfono, correo electrónico o dirección)?	○ No<br>○ Sí
Si es así, ¿cuál es la información que ha cambiado?	☐ Nuevo número de teléfono<br>☐ Nuevo correo electrónico<br>☐ Nueva dirección<br>(Marque todas las respuestas que correspondan)
Por favor de darnos su nuevo número de teléfono.	__________________<br>(xxx-xxx-xxxx)
Por favor de darnos su nuevo correo electrónico.	__________________
Por favor de darnos su nueva dirección.	______________________

Empezaremos por hacerle algunas preguntas acerca de su método anticonceptivo.

¿Qué método(s) ha usado para no quedar embarazada en las últimas 4 semanas?	☐ Implante anticonceptivo (Nexplanon)<br>☐ DIU sin hormonas de cobre (Paragard)<br>☐ DIU hormonal de 5 años (Mirena)<br>☐ DIU hormonal de 3 años (Liletta)<br>☐ Otro DIU hormonal de 3 años (Skyla)<br>☐ Inyección de 3 meses (Depo-Provera)<br>☐ Pastilla anticonceptiva (combinación hormonal)<br>☐ Pastilla anticonceptiva (solo progesterona)<br>☐ Parche anticonceptivo (Xulane)<br>☐ Anillo vaginal (NuvaRing)<br>☐ Condones masculinos<br>☐ Condones femeninos<br>☐ Capuchón vaginal o esponja<br>☐ Espermicida<br>☐ Diafragma<br>☐ Conciencia de fertilidad / Planificación familiar natural/el ritmo<br>☐ Retiro<br>☐ Anticonceptivo de emergencia Levonorgestrel (Plan B/Next Choice)<br>☐ Pastilla anticonceptiva de emergencia Ulipristal EC (Ella)<br>☐ Otro<br>☐ Ninguno; no estoy usar un método anticonceptivo<br>(Marque todas las respuestas que correspondan)
Si hay otro, por favor de especificar:	______________________________
¿Sigue usando el mismo método anticonceptivo que recibió al principio del estudio?	◯ No<br>◯ Sí
En las últimas 4 semanas, ¿ha comprobado para asegurarse que su DIU o implante todavía sigue en su lugar?	◯ No, no lo he hecho<br>◯ Sí, yo he tocado los hilos (o barra en el brazo) y confirmé que sigue ahí<br>◯ Sí, un proveedor médico confirmó los hilos<br>◯ Intenté, pero no pude sentir los hilos
¿En qué fecha le quitaron o se le cayó el aparato?<br><br>Si usted no puede recordar la fecha exacta, haga su mejor cálculo.	______________________________

¿Cuáles son las razones por las que ya no está usando el método anticonceptivo que recibió al inicio del estudio?	☐ Los problemas de sangrado (manchado , irregular, etc.)<br>☐ Demasiado sangrado<br>☐ Dolor menstrual<br>☐ Dolor<br>☐ Problemas en los senos<br>☐ Aumento de peso<br>☐ La pérdida de peso<br>☐ Mal humor o irritabilidad<br>☐ Hinchazón del abdomen<br>☐ Problemas de la piel<br>☐ Dolor durante el sexo<br>☐ Mi pareja podía sentir el DIU<br>☐ Se cayó/fue expulsado el DIU<br>☐ Quería quedar embarazada<br>☐ Tuve una prueba de embarazo positiva<br>☐ Otro<br>(Marque todas las respuestas que correspondan)
Si es otro, por favor especifique:	__________________________________

Ahora le queremos hacer algunas preguntas sobre su método anticonceptivo. Por favor piense sobre todos los métodos usted ha usado recientemente, incluyendo el DIU o el implante.

Por lo general, ¿Qué tan satisfecha está usted con el método que estaba usando durante las 4 semanas anteriores?	◯ Completamente satisfecha<br>◯ Algo satisfecha<br>◯ Ni satisfecha o insatisfecha<br>◯ Algo insatisfecha<br>◯ Completamente insatisfecha<br>◯ Prefiero no contestar esta pregunta
Por lo general, ¿qué tanta confianza le tiene al método que ha utilizado durante las últimas 4 semanas para prevenir un embarazo?	◯ Muchísima confianza<br>◯ Mucha confianza<br>◯ Algo de confianza<br>◯ Poca confianza<br>◯ Muy poca o ninguna confianza<br>◯ Prefiero no contestar esta pregunta
Por favor marque si usted está de acuerdo o en desacuerdo con la siguiente frase: "Yo siento que tengo el control sobre si o no salgo embarazada."	◯ Estoy muy de acuerdo<br>◯ Estoy un poco de acuerdo<br>◯ No estoy de acuerdo o en desacuerdo<br>◯ Estoy un poco en desacuerdo<br>◯ Estoy muy en desacuerdo<br>◯ Prefiero no contestar esta pregunta

Las últimas preguntas son sobre cualquier embarazo o problemas de salud que pudieran estar relacionados con su método anticonceptivo.

¿Ha tenido una prueba de embarazo positiva desde que contestó el último cuestionario?<br>(Eso es, en los últimos seis meses)	◯ No<br>◯ Yes<br>◯ No lo sé
¿Cuál fue la fecha de la prueba de embarazo positiva?	__________________________________
Cuando quedó embarazada, ¿estaba tratando de quedar embarazada?	◯ Si<br>◯ No

Si a usted le salió la prueba positiva, por favor de indicar el resultado del embarazo.	◯ Tuve o estoy planeando tener un aborto provocado<br>◯ Tuve un aborto espontáneo<br>◯ Tuve un embarazo ectópico<br>◯ Estoy planeando en continuar con el embarazo y criar a mi bebé<br>◯ Estoy planeando en continuar con el embarazo y dar al bebé en adopción<br>◯ No estoy segura de que voy hacer
Para garantizar su seguridad, si usted estuvo embarazada, nos gustaría saber más qué tipo de atención recibió. Por favor, indique el nombre de la clínica o del hospital donde la atendieron.	______________________
Desde que empezó el estudio, ¿ha sido hospitalizada debido a una enfermedad o una lesión?	◯ No<br>◯ Sí
Por favor de darnos la fecha:	________________
Por favor de explicarnos que pasó:	______________________
¿Ha sido atendida por un proveedor médico debido a un problema que usted pensaba que pudiera estar relacionado con el DIU o implante que le pusimos?	◯ No<br>◯ Sí
Por favor de darnos la fecha:	________________
Por favor de describir:	______________________

Gracias de nuevo por participar en este estudio. En el siguiente espacio, no dude en decirnos cualquier cosa sobre su experiencia con su método anticonceptivo o con su participación en este estudio. De lo contrario, nosotros estaremos en contacto con usted en el futuro.

Comentarios:	______________________
